# Single-cell transcriptome analysis uncovers underlying mechanisms of acute liver injury induced by tripterygium glycosides tablet in mice

**DOI:** 10.1016/j.jpha.2023.03.004

**Published:** 2023-03-22

**Authors:** Qiuyan Guo, Jiangpeng Wu, Qixin Wang, Yuwen Huang, Lin Chen, Jie Gong, Maobo Du, Guangqing Cheng, Tianming Lu, Minghong Zhao, Yuan Zhao, Chong Qiu, Fei Xia, Junzhe Zhang, Jiayun Chen, Feng Qiu, Jigang Wang

**Affiliations:** a State Key Laboratory for Quality Ensurance and Sustainable Use of Dao-di Herbs, Artemisinin Research Center, and Institute of Chinese Materia Medica, China Academy of Chinese Medical Sciences, Beijing, 100700, China; b School of Chinese Materia Medica, and State Key Laboratory of Component-Based Chinese Medicine, Tianjin University of Traditional Chinese Medicine, Tianjin, 301617, China; c Department of Nephrology, Shenzhen Key Laboratory of Kidney Diseases, Shenzhen Clinical Research Centre for Geriatrics, Shenzhen People's Hospital, The First Affiliated Hospital, Southern University of Science and Technology, Shenzhen, Guangdong, 518020, China; d College of Food Science and Engineering, Institute of Ocean, Bohai University, Jinzhou, Liaoning, 121013, China; e State Key Laboratory of Infectious Disease Prevention and Control, Collaborative Innovation Center for Diagnosis and Treatment of Infectious Diseases, National Institute for Communicable Disease Control and Prevention, Chinese Center for Disease Control and Prevention, Beijing, 102206, China; f Institute of Chinese Materia Medica, China Academy of Chinese Medical Sciences, Beijing, 100700, China; g The Fourth Clinical Medical College of Guangzhou University of Chinese Medicine, Shenzhen, Guangdong, 518033, China

**Keywords:** Tripterygium glycosides tablet, Acute liver injury, scRNA-seq

## Abstract

Tripterygium glycosides tablet (TGT), the classical commercial drug of *Tripterygium wilfordii* Hook. F. has been effectively used in the treatment of rheumatoid arthritis, nephrotic syndrome, leprosy, Behcet's syndrome, leprosy reaction and autoimmune hepatitis. However, due to its narrow and limited treatment window, TGT-induced organ toxicity (among which liver injury accounts for about 40% of clinical reports) has gained increasing attention. The present study aimed to clarify the cellular and molecular events underlying TGT-induced acute liver injury using single-cell RNA sequencing (scRNA-seq) technology. The TGT-induced acute liver injury mouse model was constructed through short-term TGT exposure and further verified by hematoxylin-eosin staining and liver function-related serum indicators, including alanine aminotransferase, aspartate aminotransferase, alkaline phosphatase and total bilirubin. Using the mouse model, we identified 15 specific subtypes of cells in the liver tissue, including endothelial cells, hepatocytes, cholangiocytes, and hepatic stellate cells. Further analysis indicated that TGT caused a significant inflammatory response in liver endothelial cells at different spatial locations; led to marked inflammatory response, apoptosis and fatty acid metabolism dysfunction in hepatocytes; activated hepatic stellate cells; brought about the activation, inflammation, and phagocytosis of liver capsular macrophages cells; resulted in immune dysfunction of liver lymphocytes; disturbed the intercellular crosstalk in liver microenvironment by regulating various signaling pathways. Thus, these findings elaborate the mechanism underlying TGT-induced acute liver injury, provide new insights into the safe and rational applications in the clinic, and complement the identification of new biomarkers and therapeutic targets for liver protection.

## Highlights

• TGT induced acute liver injury via the regulation of inflammatory response.
• TGT treatment resulted in hepatocyte apoptosis and HSC activation.
• TGT treatment caused activated LCM cells and the immune dysfunction of lymphocyte.

## Graphical abstract

**Figure:**
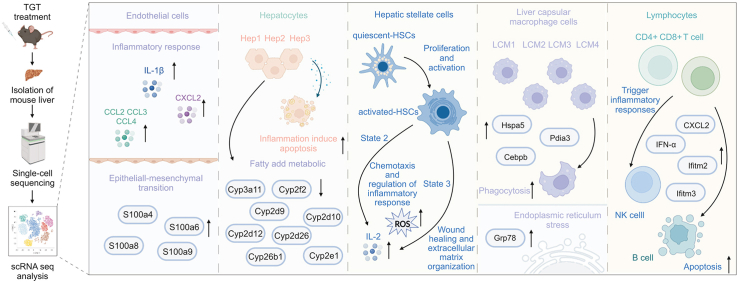

## 1 Introduction

*Tripterygium wilfordii* Hook. F. (TWHF) has been regarded as a “China-pioneered novel herbal drug” in the treatment of autoimmune and inflammatory diseases, such as rheumatoid arthritis (RA). Among the representative commercial preparations, tripterygium glycosides tablet (TGT) has similar clinical efficacy as methotrexate [1]. TGT can also be used as a therapy against nephrotic syndrome, Behcet's triad, and leprosy reaction. However, due to its narrow and limited treatment window, TGT-induced organ toxicity has drawn significant attention. Specifically, liver injury accounts for about 40% of TGT toxicity-related clinical reports [2]. The potential liver injury risk of TGT is a topic of public health interest, and extending the application of TGT by overcoming its limitations has become an area of active research [3,4].

Based on current studies, the development and application of TWHF are limited by its marked association with liver injury characterized by abnormal lipid metabolism [5]. In a previous study, we found that TGT-induced acute liver injury was partially caused by its regulation of lipid metabolism, including phosphatidylcholine, phosphatidylethanolamine, and the activity of metabolizing enzymes in the liver, such as CYP450 family members [6]. In addition, other studies have reported that TGT-induced acute liver injury is closely related to the inhibition of farnesoid X receptor signaling [7], the regulation of amino acid metabolism [8], and the suppression of peroxisome proliferator-activated receptor α (PPARα) [9]. However, the underlying cellular and molecular mechanisms of TGT-induced acute liver injury are yet to be clarified.

Single-cell RNA sequencing (scRNA-seq) is a robust tool for decomposing the transcriptomes of complex tissues at the single-cell level. In addition, it has provided a foundation for the Human Cell Atlas Project that aims to define the molecular states of all cell types in the human body [10]. As the largest internal organ in the human body, the liver performs vital functions in the metabolism of both nutrients and energy. It consists of hepatocytes that account for >60% of all liver cells and non-parenchymal cells (NPCs), including various immune cell types, hepatic stellate cells (HSCs), resident macrophages known as Kupffer cells (KCs), and liver sinusoidal endothelial cells (LSECs) [11]. Hitherto, scRNA-seq has been utilized to identify relevant cell subtypes in several liver diseases, such as chronic liver injury [12], nonalcoholic fatty liver disease [13,14], nonalcoholic steatohepatitis [15], and liver cancer [[16], [17], [18]]. This approach further deepens our understanding of TGT-induced acute liver injury pathogenesis and provides novel insights into key cellular and molecular mechanisms underlying liver repair, thereby enabling the identification of critical targets for patients across the spectrum of TGT-induced acute liver injury.

In the present study, we employed scRNA-seq technology to dissect the alterations in liver gene expression and functional signaling pathways after TGT treatment. The changes in gene expression and modulations of different cell types in the liver tissue were elucidated to delve into the liver microenvironment that is substantially remodeled by TGT. Thus, we speculated that our findings provide novel insights into the cellular and molecular mechanisms of TGT-induced acute liver injury and complement the further identification of potential therapeutic targets to alleviate the burden of TGT toxicity.

## 2 Materials and methods

### 2.1 Mice and treatments

The experiments on C57BL/6 mice were in line with the regulations of the Institutional Animal Care and Use Committee and Animal Ethics Committee of the Institute of Chinese Materia Medica (Approval number: 2022B106). Male C57BL/6 mice (8-weeks-old) were bought from Beijing Vital River Laboratory Animal Technology Co., Ltd (Beijing, China). Mice were kept under specific pathogen-free (SPF) conditions and acclimatized for a week before randomly dividing into two groups (6 mice/group). The animals were administered normal saline or TGT Provided by Zhejiang DND Pharmaceutical Co., Ltd (Product code number: Z33020422, Xinchang, China) at a concentration of 270 mg/kg (which is about 20-fold of the clinical daily dose) intragastrically, 18 h before they were sacrificed.

### 2.2 Serum samples detection

The serum concentrations of alanine aminotransferase (ALT), aspartate aminotransferase (AST), alkaline phosphatase (AKP), and total bilirubin (TBIL) were detected using the corresponding kits, according to manufacturer's instructions (Product batch numbers: 20211111, 20211110, 20211108, and 20211111; Nanjing Jiancheng Bioengineering Institute, Nanjing, China). The serum inflammatory factors interleukin (IL)-1β, IL-2, CXCL2, CCL2, CCL3, CCL4, and interferon (IFN)-α were detected by enzyme-linked immunosorbent assay (ELISA) kit (Product batch numbers: E20221114-20174A, E20221114-20176A, E20221114-21268A, E20221114-22324A, E20221114-20427A, E20221114-20431A, and E20221114-20113A; Shanghai Enzyme-linked Biotechnology Co., Ltd., Shanghai, China).

### 2.3 Hematoxylin-eosin (HE) and immunofluorescence staining

Mouse liver tissues were fixed in 4% paraformaldehyde, dehydrated, embedded in paraffin, and cut into 5-μm-thick sections before HE staining for pathological observation. The images were captured through a microscope (DM5000B, Leica, Wetzlar, Germany) at × 40 magnification.

The liver sections were incubated with S100 calcium binding protein A8 (*S100a8*) and glucose regulated protein 78 (*Grp78*) antibodies at 4 °C for 12 h, respectively, washed three times with Tris-Buffered Saline and Tween 20 (TBST), and incubated with secondary antibodies at room temperature on a shaker for 1 h. Subsequently, the sections were washed with TBST, stained with 4′,6-diamidino-2-phenylindole (DAPI) for 5 min, and covered with an anti-fading fixative, and fluorescence images were captured.

### 2.4 Single-cell isolation

The mouse liver tissues from the control and TGT groups were collected for scRNA-seq analysis. Briefly, two liver samples from each group were dissected, sliced into small pieces, and enzymatically digested using the Liver Dissociation Kit for mouse (130-105-807, MiltenyiBiotec, Bergisch Gladbach, Germany) for about 30 min, according to the manufacturer's protocol. Then, the dissociated cells were filtered through a 70-mm strainer and rinsed with 15 mL Dulbecco's modified Eagle medium (DMEM) until a single-cell suspension was obtained. Subsequently, the cells were collected by centrifugation at 300 *g* for 10 min before resuspension in 1 mL phosphate-buffered saline (PBS). The red blood cells were removed using the Red Blood Cell Lysis Solution (130-094-183, MiltenyiBiotec). The above samples were washed twice with PBS for single-cell suspension.

### 2.5 Single-cell RNA-seq

The single-cell suspensions collected from the above step were used for scRNA-seq library construction following the instruction of the Single Cell 3′ Reagent Kit v3.1 (10× Genomics, PN-1000121, Pleasanton, CA, USA). The scRNA-seq libraries were constructed using the Chromium Next GEM Single Cell 3′ Kit v3.1 from 10× Genomics, according to the manufacturer's instructions. Briefly, single cells were diluted to a final concentration of 1,000 cells/μL. Then, approximately 10,000 cells were captured in droplets to generate nanoliter-scale Gel beads in EMulsion (GEMs). Then, GEMs were reverse transcribed before cell barcoding, the emulsions were broken, and the cDNA was separated and further purified before PCR amplification. Next, the obtained amplified cDNA was used for library construction. To build-up the RNA-seq library, the amplified cDNA was fragmented and end-repaired, followed by double-sided size selection and PCR amplification with sample index primers. The libraries were further purified and profiled for quality evaluation and sequenced on the Illumina NovaSeq 6000 (San Diego, CA, USA) platform.

### 2.6 scRNA-seq data pretreatment

The raw gene expression matrix was produced by the Cell ranger (version 6.0.1) pipeline along with the mouse reference genome (mm10) and analyzed with the Seurat R package (version 4.0.3). The low-quality cells were filtered by the sample-specific criteria due to different data qualities, and the remaining high-quality cells were selected for further analysis. Seurat's SCTransform function was used for the normalization and scaling of the samples. We integrated high-quality cells into a matrix and performed cell clustering based on common features. The last step was scRNA-seq visualization and analysis of all the clustered cells by uniform manifold approximation and projection (UMAP) algorithm.

### 2.7 Cell type identification

Different types of cells were identified by the expression levels of known canonical markers in each cluster using Seurat's FindAllMarkers function. The cell subtype identification was confirmed by principal components analysis (PCA), clustering, and annotation.

### 2.8 Differentially expressed genes (DEGs) and gene functional enrichment analysis

The DEG analysis for each cell type between the control and TGT groups was conducted by the Seurat FindMarkers function with the selection standard of |avg_log2FC| > 0.25, min.pct > 0.1, and adjusted *P*-value < 0.05. The violin plots or heatmap using Seurat VlnPlot function and R packages pheatmap (v1.0.12) were generated for further visualization of markers. Gene Ontology (GO) analysis was conducted with the clusterProfiler R package (version 3.18.1) based on the up- and down-regulated genes among DEGs between different groups. The pathway enrichment analysis with adjusted *P*-value < 0.05 was further visualized by the R package ggplot2 (v3.3.3).

### 2.9 Pseudotime analysis

To investigate the cell-state transitions of hepatocytes and HSCs, we performed pseudo-temporal analysis by the Monocle2 R package (version 2.18.0). Briefly, we converted Seurat object to CDS object, followed by the confirmation of differential cell states based on the identification of significantly changed genes by the differential GeneTest function. Subsequently, the plot lineage trajectories were performed using the plot_cell trajectory function.

### 2.10 Ligand-receptor interaction analysis

The ligand-receptor interaction analysis was performed using CellChat R package (version 1.1.3) with the recommended parameters. The tool first utilized the normalized gene expression data and cell type meta information from the TGT and control groups to generate different CellChat objects. The two objects were merged using the mergeCellChat function for subsequent analysis. The communication probability value and permutation test were calculated in the ligand-receptor pairs of all cell types between the TGT and control groups. Results with a *P*-value < 0.05 were considered significant ligand-receptor pairs. The results were displayed by bubble plots and chord diagrams.

### 2.11 Statistical analysis

In this study, results are presented as mean ± standard error of the mean. One-way analysis of variance (ANOVA) was used to analyze the statistical differences between the control and TGT groups. The evaluation of gene set scores with *P*-value < 0.05 (^∗^*P* < 0.05, ^∗^^∗^*P* < 0.01, ^∗∗^^∗^*P* < 0.001) was considered significant in the Seurat's AddModuleScore function.

## 3 Results

### 3.1 TGT significantly altered the body weight, liver morphology, organ index, and serum ALT, AST, AKP, and TBIL levels of mice

**Fig. 1:**
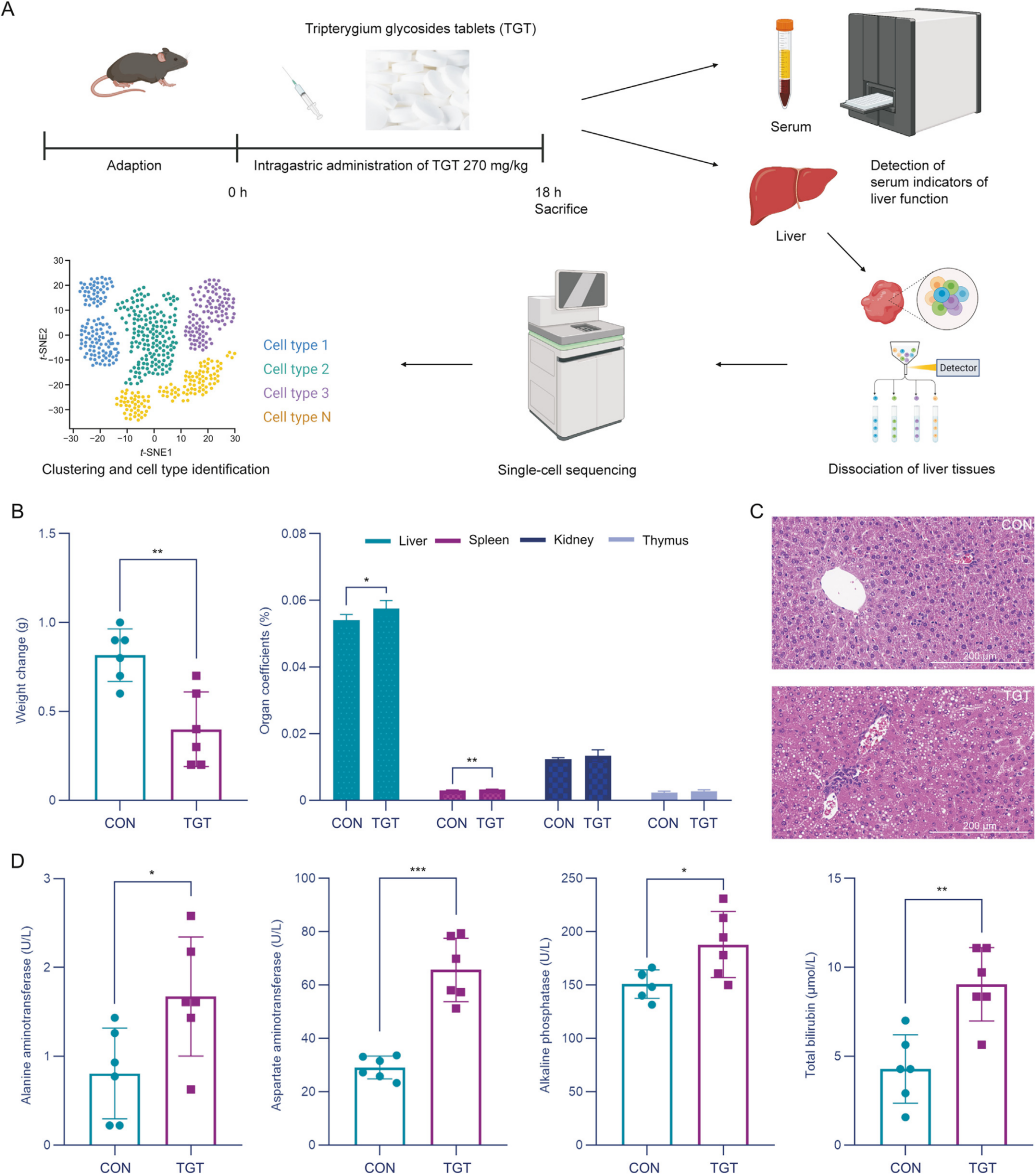
Influence of tripterygium glycosides tablet (TGT) on the organ index, liver histopathology, and serum biochemical parameters. (A) Schematic of the TGT animal experiments. (B) Effects of TGT on mouse weight and organ coefficients, *n* = 6. (C) Representative hematoxylin-eosin (HE) staining of livers for histological examination (40×). (D) Effects of TGT on alanine aminotransferase, aspartate aminotransferase, alkaline phosphatase, and total bilirubin in mouse serum, *n* = 6. Data are expressed as the mean ± standard deviation (SD); ^∗^*P* < 0.05, ^∗∗^*P* < 0.01, ^∗∗∗^*P* < 0.001 vs*.* CON. CON: Control.

A TGT-induced acute liver injury mouse model was constructed and evaluated with serum and organ indices before performing single-cell sequencing and cell type identification (Fig. 1A). According to the body weight changes of mice in different groups, TGT treatment markedly reduced the body weight of mice compared to the control group (Fig. 1B). Moreover, organ coefficients demonstrated that TGT significantly increased the liver weight while decreasing the spleen weight of mice and did not show any marked influence on the kidneys and thymus. The above data indicated that TGT treatment might lead to hepatomegaly and immune dysfunction, with the need to be further validated. As described in Fig. 1C, TGT treatment markedly changed the liver morphology of mice compared to the control group. Specifically, mice in the control group showed healthy liver tissue with clear hepatic lobules and well-arranged hepatocytes, whereas TGT administration caused acute liver injury with features of disappeared live basic structure, obvious steatosis, and severe inflammatory cell infiltration. Based on the serum concentration results of ALT, AST, AKP, and TBIL in comparison with the control, mice in the TGT-induced acute liver injury group showed high concentrations of the above serum biochemical parameters (all *P* < 0.05, Fig. 1D).

### 3.2 Single-cell profiling of the liver landscape under TGT-induced acute liver injury conditions

**Fig. 2:**
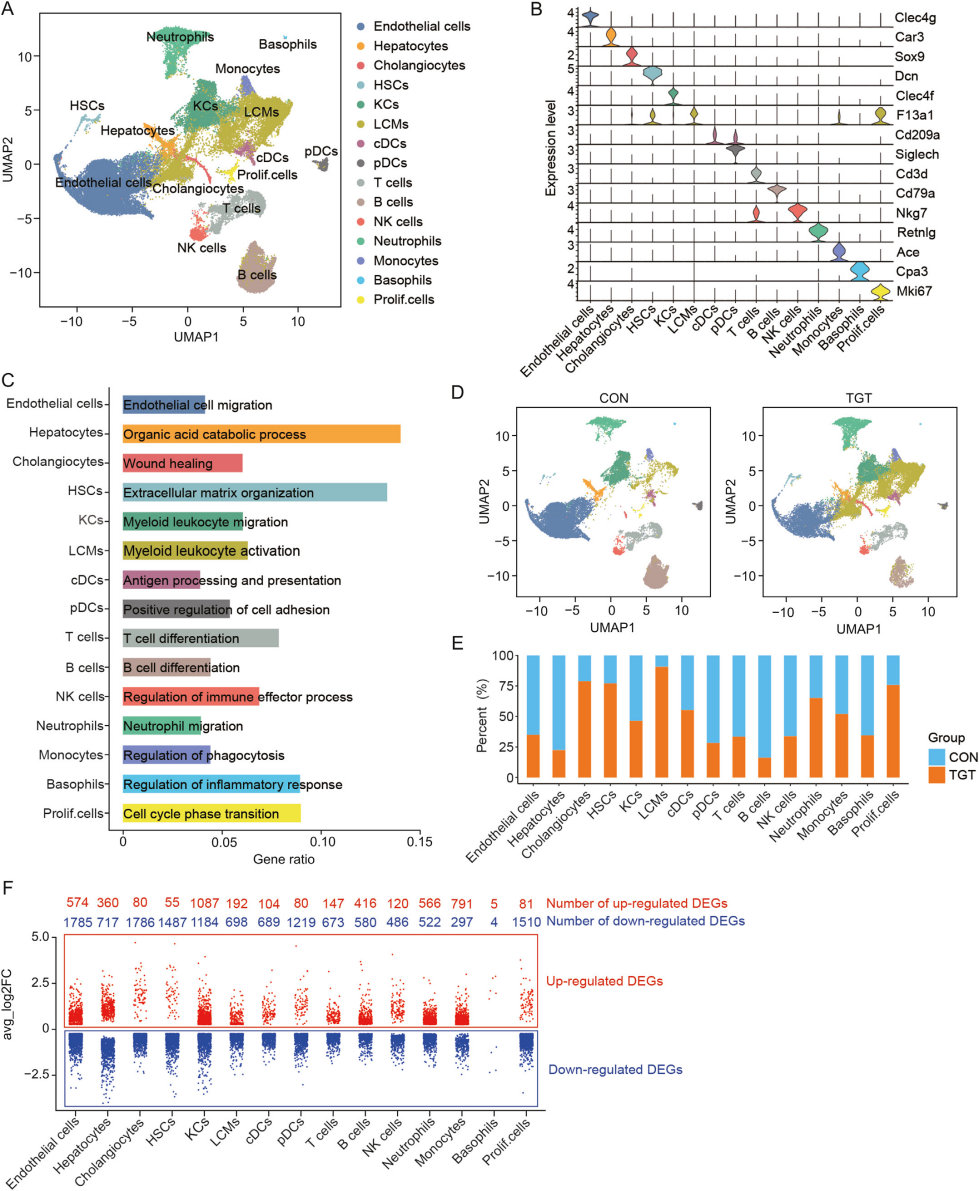
Tripterygium glycosides tablet (TGT) exposure alters the single-cell RNA sequencing (scRNA-seq) profiling of mice liver tissues. (A) Uniform manifold approximation and projection (UMAP) displays 15 distinct cell clusters. (B) Violin plot shows the expression of marker genes for each cell type. (C) Functional enrichment of each cell type. (D) Distribution comparison of clusters from control (CON) and TGT groups. (E) Percent contribution of CON (blue) and TGT (orange) mouse liver cells for each cell type. (F) Distributions of up- and down-regulated genes for each cell type after TGT treatment. HSC: hepatic stellate cell; KC: Kupffer cell; cDC: conventional dendritic cell; pDC: plasmacytoid dendritic cell; NK: natural killer; DEG: differentially expressed gene*.*

To explore the cellular and molecular mechanisms underlying TGT-induced acute liver injury, we performed the single-cell transcriptional sequencing of livers from both the control and TGT-treated mice (*n* = 2) with 10× Genomics technology. After quality control (Figs. S1A–C), 46,688 cells were analyzed (23,535 for the control group and 23,153 for TGT treatment) for further identification of cell type, as shown in Fig. 2. Consequently, 15 cell types (Fig. 2A) were identified in the mouse liver tissues based on the canonical cell markers (Fig. 2B), including endothelial cells (*n* = 14,408, expressing *Clec4g*), hepatocytes (*n* = 1,051, expressing *Car3*), cholangiocytes (*n* = 459, expressing *Sox9*), HSCs (*n* = 280, expressing *Dcn*, and *F13a1*), Kupffer cells (KCs, *n* = 5,147, expressing *Clec4f*), liver capsular macrophages (LCMs, *n* = 10,184, expressing *F13a1*), conventional dendritic cells (cDC, *n* = 697, expressing *Cd209a*), plasmacytoid dendritic cells (pDC, *n* = 672, expressing *Siglech*), T cells (*n* = 3,111, expressing *Cd3d*), B cells (*n* = 5,251, expressing *Cd79a*), natural killer cells (NK, *n* = 817, expressing *Nkg7*), neutrophils (*n* = 3,578, expressing *Retnlg*), monocytes (*n* = 609, expressing *Ace*), basophils (*n* = 81, *Cpa3*), and Prolif.cells (*n* = 343, expressing *Mki67*). Further function enrichment indicated that endothelial cells were involved in endothelial cell migration, hepatocytes in the organic acid catabolic process, HSCs in the extracellular matrix organization, T/B cells in cell differentiation, and LCMs in myeloid leukocyte activation (Fig. 2C). TGT treatment led to marked changes of cell numbers in all 15 cell types in the liver (Figs. 2D, 2E, S1D and S1E). Specifically, the relative percentages of cholangiocytes, HSCs, and LCMs significantly increased after TGT treatment, while the proportion of endothelial cells, hepatocytes, and lymphocytes (including T cells, NK cells, and B cells) decreased. In addition, TGT treatment significantly altered the gene expression profiles (up- and down-regulated genes) in the above 15 cell types. Endothelial cells displayed significant DEG after TGT treatment, including 574 up- and 1,785 down-regulated genes (Fig. 2F), leading us to focus on endothelial cells for subsequent analysis.

### 3.3 TGT treatment led to significant inflammatory response in liver endothelial cells at different spatial locations

**Fig. 3:**
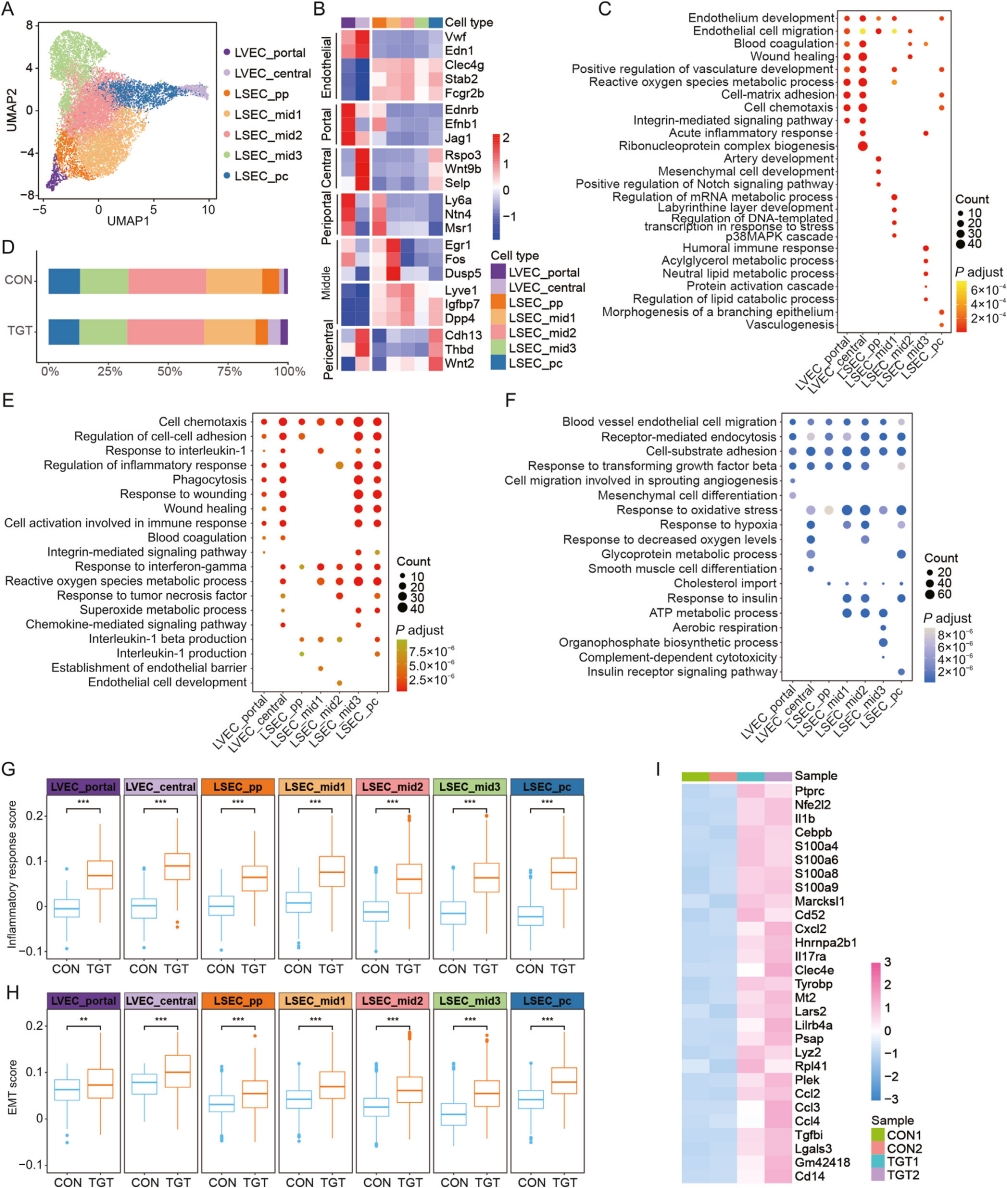
Endothelial cells at different spatial locations undergo inflammatory response. (A) The uniform manifold approximation and projection (UMAP) displaying the subtypes of endothelial cells based on spatial locations. (B) The heatmap plot showing the expression of marker genes for each endothelial subtype at different spatial locations. (C) The bubble plot represents the Gene Ontology (GO) enrichment analysis for every endothelial cell cluster. (D) The bar plot showing the fraction of endothelial cell subtypes in control (CON) and tripterygium glycosides tablet (TGT) groups. (E) The bubble plot represents the GO enrichment analysis of up-regulated DEGs in whole endothelial cell subtypes. (F) The bubble plot represents the GO enrichment analysis of down-regulated DEGs in whole endothelial cell subtypes. (G) The functional comparisons of inflammatory response scores for each cluster between CON and TGT groups. ^∗∗∗^*P* < 0.001. (H) The functional comparisons of epithelial-mesenchymal transition (EMT) scores for each cluster between CON and TGT groups. ^∗∗^*P* < 0.01, ^∗∗∗^*P* < 0.001. (I) The heatmap plot showing common up-regulated differentially expressed genes (DEGs) for all endothelial cells (ECs) clusters. LVEC: liver vascular endothelial cell; LSEC: liver sinusoidal endothelial cell.

Liver endothelial cells consist of major cell types, including liver vascular endothelial cells (LVECs) and liver sinusoidal endothelial cells (LSECs), and play a major role in liver functions, such as liver regeneration and fibrosis under both physiological and pathological conditions [19]. As shown in Fig. 3A, we identified the expression patterns of seven subtypes of endothelial cells (two LVEC subtypes and five LSEC subtypes) based on spatial locations of different landmark genes. For example, LVEC_portal expressed *Ednrb*, *Efnb1*, and *Jag1*, whereas LVEC_central expressed *Rspo3*, *Wnt9b*, and *Selp* (Fig. 3B). Among these subtypes, functional annotations indicated the involvement of the two LVEC subtypes in “endothelium development,” “wound healing,” “positive regulation of vasculature development,” “cell-matrix adhesion,” and “cell chemotaxis.” (Fig. 3C). Notably, the LSEC subtypes exhibited decreased proportions (especially LSEC_pp), while the LVEC_central and LVEC_portal subtypes showed markedly increased proportions after TGT treatment (Figs. 3D, S2A, S2B and S2C). In addition, the upregulated genes after TGT administration among liver endothelial cells were enriched in cell chemotaxis, regulation of inflammatory response, and cell activation involved in immune response (Fig. 3E), while the downregulated genes were enriched in cell-substrate adhesion and response to oxidative stress-related signaling pathways (Fig. 3F). To further validate the influence of TGT on liver endothelial cells, we calculated the inflammatory response scores of the seven cell subtypes between the control and TGT groups. TGT administration significantly increased the inflammatory response score compared to the control group (all *P* < 0.001, Figs. 3G and S2D). We also observed that some key proinflammatory cytokines, including *IL-1β*, *CXCL2*, *CCL2*, *CCL3*, and *CCL4*, were significantly overexpressed in all endothelial cell subtypes after TGT stimulation (Figs. 3H, and S2E). These cytokines are responsible for the recruitment of immune cells to the location of inflammation. Epithelial-mesenchymal transition (EMT) affected by sustained inflammation is closely associated with liver fibrosis and regeneration by enhancing the cellular ability of metastasis and invasion. Based on our findings, TGT administration markedly increases the EMT score in all the above seven cell subtypes (all *P* < 0.01, Figs. 3I, and S2F). Further analysis indicated that TGT treatment prompted the endothelial cells at different spatial locations to undergo inflammatory responses by upregulating the following genes: *IL-1*β, *CXCL2*, *CCL2*, *CCL3*, and *CCL4* (Fig. 3I, and Fig. S2D). Moreover, the expression of several *S100* isoforms (including *S100a4*, *S100a6*, *S100a8*, and *S100a9*) has increased pro-inflammatory and pro-fibrotic activities [20] in all endothelial cell subtypes under TGT stimulation.

### 3.4 TGT treatment caused marked inflammatory response, apoptosis, and fatty acid metabolism dysfunction in hepatocytes

**Fig. 4:**
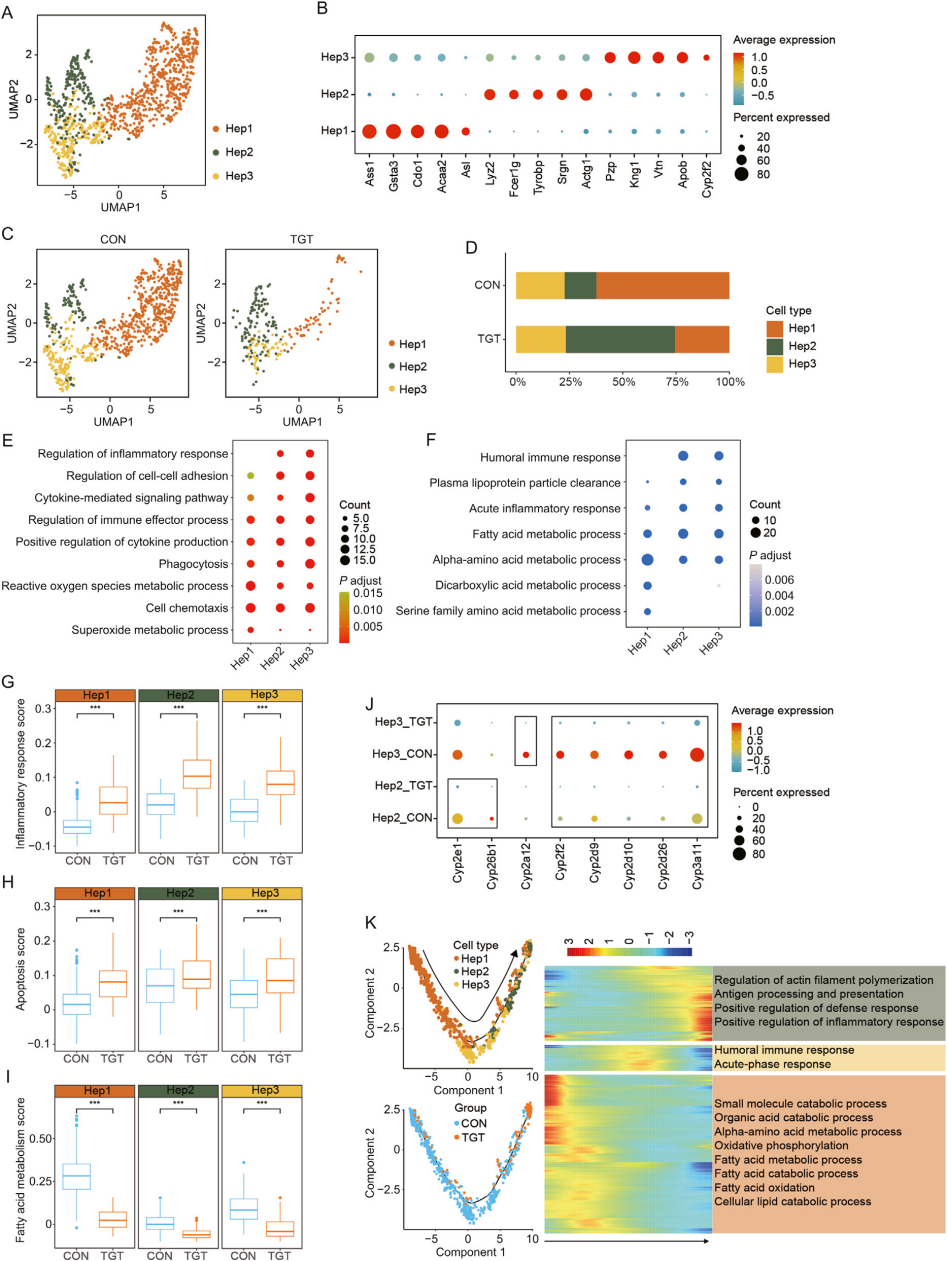
Heterogeneity of hepatocytes and their differentiation states response to tripterygium glycosides tablet (TGT). (A) The uniform manifold approximation and projection (UMAP) displaying the subtypes of hepatocytes. (B) The dot plots illustrating the expression of subtype-specific genes for each hepatocyte subtype. (C) Distribution comparison of hepatocyte subtypes from control (CON) and TGT groups. (D) The bar plot showing the fraction of hepatocyte subtypes in CON and TGT groups. (E) The bubble plot represents the Gene Ontology (GO) enrichment analysis of up-regulated differentially expressed genes (DEGs) in whole hepatocyte subtypes. (F) The bubble plot represents the GO enrichment analysis of down-regulated DEGs in whole hepatocyte subtypes. (G–I) The functional comparisons of inflammatory response scores (G), apoptosis scores (H), and fatty acid metabolism scores (I) for each cluster between CON and TGT groups. ^∗∗∗^*P* < 0.001. (J) The differentially expressed cytochrome P450 enzymes (CYPs) in Hep2 and Hep3 between control and TGT groups. (K) Pseudotime trajectory analysis of hepatocyte subtypes based on cell subtypes and group.

As shown previously, the cell number of hepatocytes was markedly decreased after TGT administration. Next, we investigated the heterogeneity of hepatocytes and their differentiation states in response to TGT. Three subtypes of hepatocytes were identified: Hep1, Hep2, and Hep3 (Fig. 4A). The expression of subtype-specific genes varied for each hepatocyte subtype. In detail, Hep1 is characterized by the expression of *Ass1*, *Gsta3*, *Cdo1*, *Acaa2*, and *Asl*, Hep2 by *Lyz2*, *Fcer1g*, *Tyrobp*, *Srgn*, and *Actg1*, and Hep3 by *Pzp*, *Kng1*, *Vtn*, *Apob*, and *Cyp2f2* (Figs. 4B, and S3A). The three subtypes performed different functions in response to TGT treatment. Hep1 was mainly responsible for fatty acid and amino acid metabolism, Hep2 focused mainly on cell chemotaxis and wound healing, and Hep3 was related to immune responses and processes (Fig. S3B). In contrast to the control group, TGT significantly decreased the fraction of Hep1, whereas it increased that of Hep2 (Figs. 4C, 4D, S3C, and S3D). In comparison to the control group, the upregulated DEGs were closely involved in inflammation and immune-related pathways, such as cytokine-mediated signaling pathway, regulation of immune effector process, positive regulation of cytokine production, and cell chemotaxis among all the three subtypes of hepatocytes (Fig. 4E). On the other hand, after TGT treatment, the downregulated DEGs were mainly enriched in the acute inflammatory response, fatty acid metabolic process, and alpha-amino acid metabolic process-related pathways among all subtypes of hepatocytes (Fig. 4F). Further analysis indicated that TGT treatment significantly increased the inflammatory response score (Figs. 4G, and S3E) and apoptosis score (Figs. 4H, and S3F) but decreased the fatty acid metabolism score (Figs. 4I, and S3G) among all hepatocyte subtypes (all *P* < 0.001). Moreover, one of the mechanisms of TGT-induced hepatotoxicity is to inhibit the expression of cytochrome P450 enzymes (CYPs), thereby reducing the metabolic capacity of the liver. In this study, we observed that *Cyp3a11*, *Cyp2f2*, *Cyp2d9*, *Cyp2d10*, and *Cyp2d26* were significantly decreased in Hep2 and Hep3 of the TGT group. Interestingly, *Cyp2e1* and *Cyp26b1* were significantly reduced only in Hep2 and *Cyp2a12* only in Hep3 (Fig. 4J). The transcriptional dynamics of hepatocytes during TGT administration was demonstrated by pseudotime trajectory analysis through reconstructing cell-cell correlation. As shown in Fig. 4K, the trajectory started with Hep1 cells, and most Hep3 cells progressed to the Hep2 subtype (Fig. S3H). Moreover, the trajectory began with the normal control state and ended with the TGT state, and the fraction of different states was in accordance with the functional enrichment results (Figs. 4K, and S3I). To confirm the function of Hep2 in response to TGT treatment, we identified the unique upregulated genes of Hep2, including *S100a8*, *S100a9*, and *Clec4e* (Fig. S3J). These genes have already been confirmed to play a decisive role in the development of inflammation [[21], [22], [23]]. Additionally, a systematic comparison was performed by examining the gene expression patterns in the three subtypes of hepatocytes with respect to the human hepatocyte clusters (Fig. S3K) [24]. Hep2 showed a weak correlation with Hepatocyte_3 with numerous active immune pathways, and Hepatocyte_3 correlated with drug metabolism and Wnt activation, supporting a pivotal role of Hep2 cells in response to TGT stimulation. In summary, TGT treatment induced the apoptosis of hepatocytes and positively regulated inflammation.

### 3.5 TGT treatment significantly activated HSCs

**Fig. 5:**
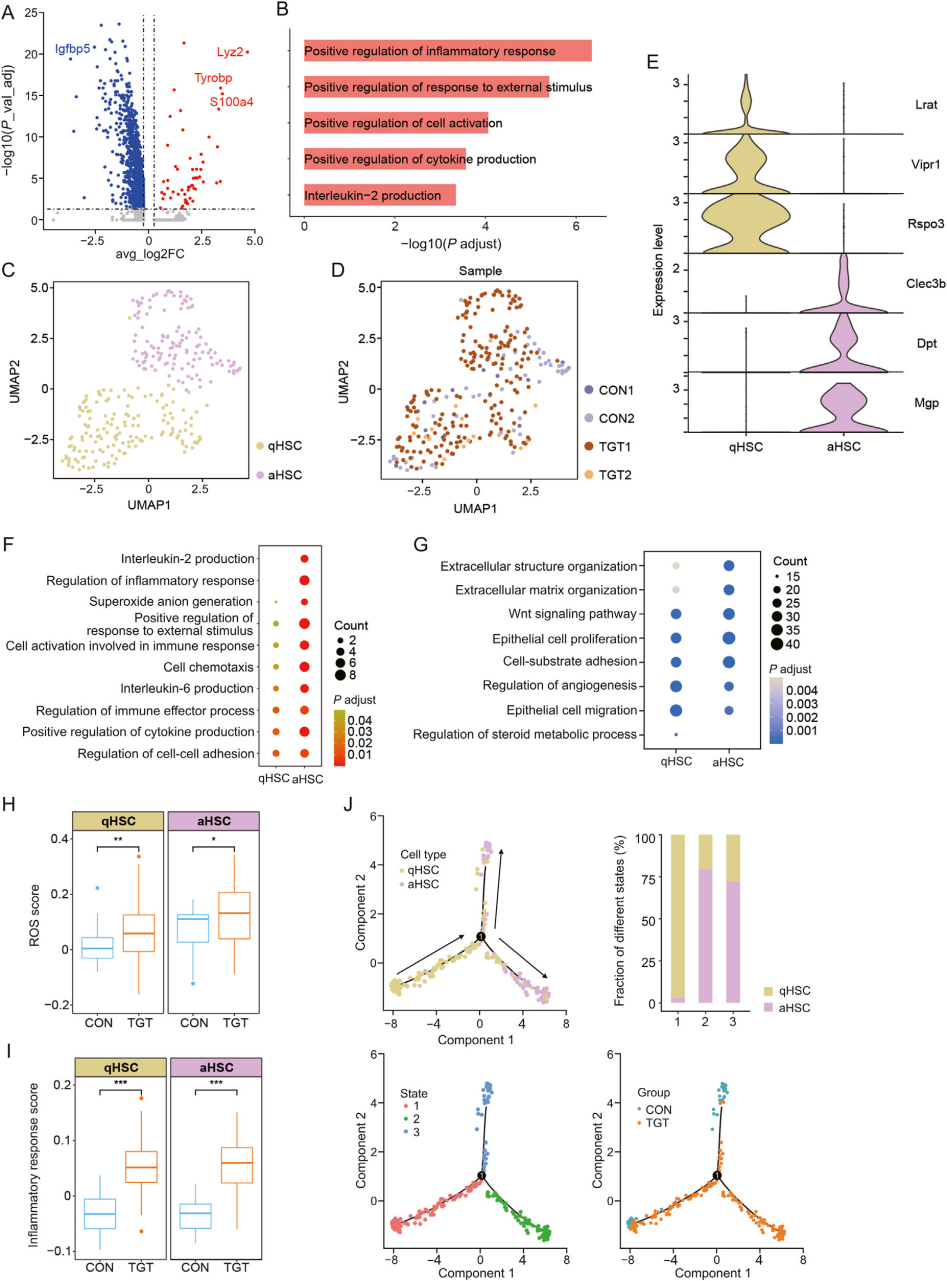
Hepatic stellate cells (HSCs) activation after tripterygium glycosides tablet (TGT) stimulation. (A) The volcano plot displaying up-regulated and down-regulated differentially expressed genes (DEGs) between control and TGT-treated HSCs. (B) Gene Ontology (GO) analysis for up-regulated DEGs of total HSCs after TGT treatment. (C) The uniform manifold approximation and projection (UMAP) showing the two subtypes of HSCs including quiescent subtype (qHSC) and the activated subtype (aHSC). (D) The UMAP showing the sample distribution corresponding to the identified cell subtypes. (E) The violin plot illustrating the expression of subtype-specific genes for each HSC subtype. (F) The bubble plot represents the GO enrichment analysis of up-regulated DEGs in whole HSC subtypes. (G) The bubble plot represents the GO enrichment analysis of down-regulated DEGs in whole HSC subtypes. (H) The functional comparisons of reactive oxygen species (ROS) scores for each cluster between control (CON) and TGT groups. ^∗^*P* < 0.05, ^∗∗^*P* < 0.01. (I) The functional comparisons of inflammatory response scores for each cluster between CON and TGT groups. ^∗∗∗^*P* < 0.001. (J) Pseudotime trajectory analysis of HSC subtypes based on cell subtypes, group and cell state.

HSCs were significantly increased after TGT treatment under liver injury conditions. Based on the current analysis, HSCs expressed different genes between the TGT and normal groups, wherein the major downregulated DEG is *Igfbp5*, while *Lyz2*, *Tyrobp*, and *S100a4* are the main upregulated DEGs (Fig. 5A). Further general function enrichment indicated that the DEGs were mainly involved in inflammation-related pathways, including the positive regulation of inflammatory response/response to external stimulus/cell activation/cytokine production and interleukin-2 (IL-2) production (Fig. 5B). UMAP showed that HSCs were divided into two subtypes, the quiescent subtype (qHSC) and the activated subtype (aHSC) (Figs. 5C and D) identified by the significantly different expression levels of *Lrat*/*Vipr1*/*Rspo3* and *Clec3b*/*Dpt*/*Mgp*, respectively (Figs. 5E, and S4A). The activation of HSCs is regarded as a major driver of liver injury. In this study, we found that TGT could induce the proliferation and activation of HSCs (Fig. S4B). The subsequent GO enrichment analysis of upregulated DEGs in whole HSC subtypes showed that compared to qHSC, aHSC overexpressed genes produced IL-2 and regulated inflammatory response (Fig. 5F). Conversely, the downregulated DEGs in whole HSC subtypes demonstrated that both subtypes decreased the expression of genes involved in epithelial cell proliferation/migration as well as cell-substrate adhesion and regulation of angiogenesis, especially qHSC genes that participated in the regulation of steroid metabolic process (Fig. 5G). In contrast to the control group, we discovered that TGT treatment significantly increased reactive oxygen species (ROS) scores and inflammatory response scores in both qHSC and aHSC subtypes (Figs. 5H, 5I, S4C, and S4D). Moreover, the trajectory of HSCs started with qHSC and ended with aHSC, and the fraction of different states was in accordance with the functional enrichment results (Figs. 5J, S4E, and S4F). Strikingly, aHSC transferred two different states, wherein state 2 (cell fate 1) was involved in cell chemotaxis and regulation of inflammatory response and state 3 (cell fate 2) in wound healing and extracellular matrix organization (Fig. S4G). On the other hand, the initial normal control state changed to the TGT state, as described in Fig. 5J. The above findings revealed that TGT treatment enhances the progress of HSCs from quiescent into an activated subtype, overexpressing the inflammatory genes while reducing the expression of genes associated with epithelial cell proliferation/migration, cell substrate adhesion, and regulation of angiogenesis.

### 3.6 TGT treatment led to the activation, inflammation, and phagocytosis of LCM cells

**Fig. 6:**
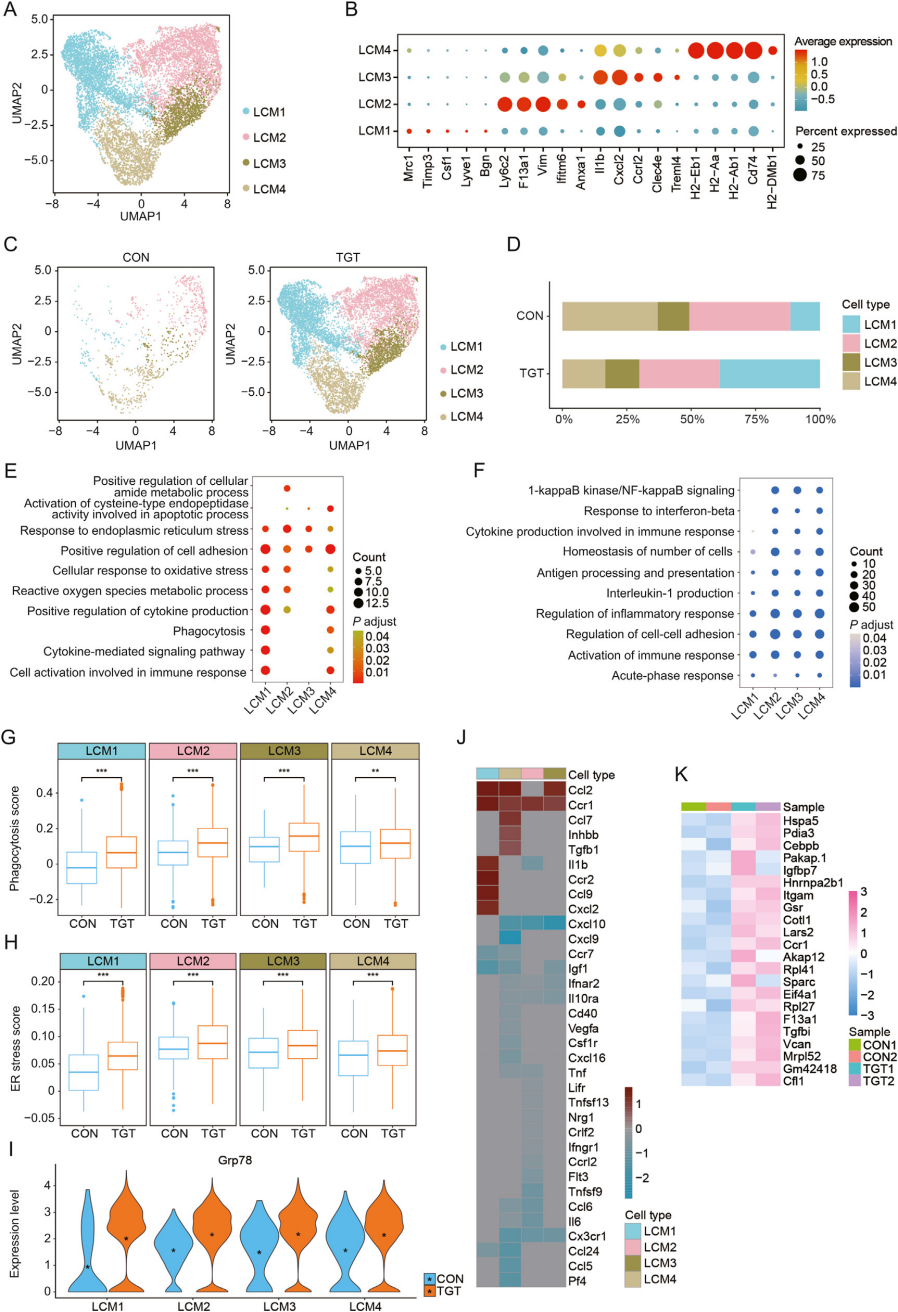
Activated liver capsular macrophage (LCM) cells induce liver damage in the tripterygium glycosides tablet (TGT) group. (A) Uniform manifold approximation and projection (UMAP) displays the subtypes of LCM cells. (B) Dot plots illustrate the expression of subtype-specific genes for each LCM cell subtype. (C) Distribution comparison of LCM cell subtypes from control (CON) and TGT groups. (D) Bar plot showing the fraction of LCM cell subtypes in the CON and TGT groups. (E) Bubble plot represents the Gene Ontology (GO) enrichment analysis of upregulated differentially expressed genes (DEGs) in whole LCM cell subtypes. (F) Bubble plot represents the GO enrichment analysis of downregulated DEGs in whole LCM cell subtypes. (G) Functional comparisons of phagocytosis scores for each cluster between the CON and TGT groups. ^∗∗^*P* < 0.01, ^∗∗∗^*P* < 0.001. (H) Functional comparisons of endoplasmic reticulum (ER) stress scores for each cluster between the CON and TGT groups. ^∗∗∗^*P* < 0.001. (I) The violin plots show the expression of *Grp78* gene for all cell subtypes in the two groups. (J) Differentially expressed cytokines for each LCM cell subtype between the control and TGT groups. Red indicates positive avg_log2FC, blue indicates negative avg_log2FC, and gray indicates no change. (K) The heatmap plot shows common upregulated DEGs for all LCM clusters.

LCMs are major cellular components of the liver, which serve critical functions in the regulation of both liver health and injury responses [25]. In this study, LCM cells were divided by gene markers into four subtypes, including LCM1, LCM2, LCM3, and LCM4, as described in Fig. 6A. LCM1 was characterized by the expression of *Mrc1*, *Timp3*, *Csf1*, *Lyve1*, and *Bgn*, LCM2 by *Ly6c2*, *F13a1*, *Vim*, *Ifitm6*, and *Anxa1*, LCM3 by *IL-1b*, *CXCL2*, *CCRL2*, and *Treml4*, and LCM4 by *H2-Eb1*, *H2-Aa*, *H2-Ab1*, *H2−DMb1*, and *Cd47* (Figs. 6B, and S5A). As shown in Figs. 6C and D, the fraction of the above four subtypes was different between the control and TGT treatment groups (Figs. S5B and C). Among these, the fraction of LCM1 was significantly increased, whereas that of LCM4 was markedly decreased. The GO analysis for upregulated DEGs indicated that LCM1 cells are enriched in the positive regulation of cell adhesion/cytokine production, cytokine-mediated signaling pathway, and cell activation involved in immune response; LCM2 cells regulate the response to endoplasmic reticulum stress, cellular response to oxidative stress, and positive regulation of cellular amide metabolism; LCM3 cells are enriched in the positive regulation of cell adhesion and response to endoplasmic reticulum stress; LCM4 cells participate in the activation of cysteine-type endopeptidase activity in apoptotic process, positive regulation of cell adhesion/cytokine production, and cell activation in immune response (Fig. 6E). Similarly, the GO analysis for downregulated DEGs for LCM cells demonstrated that the four subtypes are closely enriched in the regulation of inflammatory response/cell-cell adhesion and activation of immune response (Fig. 6F). In addition, we calculated the phagocytosis score (Figs. 6G, and S5D) and endoplasmic reticulum (ER) stress score (Figs. 6H, and S5E) of both groups in the above four subtypes. Compared to the control group, TGT treatment significantly increased the phagocytosis and ER stress scores in all the subtypes of LCM cells. Moreover, the expression level of the *Grp78* gene was significantly increased after TGT treatment in all the LCM cell subtypes (Fig. 6I). *Grp78*, also known as *BiP* and *HSP5a*, plays a critical role in the regulation of unfolded protein response (UPR) after activating ER stress in the cells. Due to the regulatory effects of LCM cells on cytokines, we analyzed the differentially expressed cytokines of all LCM cell subtypes. In the TGT group, LCM1 cells had a higher expression of *CCL2*, *CCR1*, *IL-1b*, *CCR2*, *CCL9*, and *CXCL2*, but lower expression of *CCR7* and *IGF-1*; LCM2 cells had a higher expression of *CCL2*, *CCR*1, *CCL7*, *Inhbb*, and *Tgfb1*, but lower expression of *CXCL10*, *CXCL9*, *CX3CR1*, *CCL24*, *CCL5*, and *Pf4*; LCM3 cells had a higher expression of *CCR1*, but lower expression of *IL-1b*, *CXCL10*, *Tnfsf9*, *CCL6*, *IL-6*, and *CX3CR1*; LCM4 cells had a higher expression of *CCL2* and *CCR1*, but lower expression of *CXCL10*, *IGF-1*, *Ifnar2*, *IL-*10RA, and *CX3CR1* (Fig. 6J). Among all the DEGs between the control and TGT treatment groups for all LCMs, we identified 22 common upregulated DEGs which responded to endoplasmic reticulum stress and proliferation, including *Hspa5*, *Pdia3*, and *Cebpb* (Figs. 6K, S5F, and S5G). In summary, the TGT treatment led to the activation, inflammation, and phagocytosis of LCM cells.

### 3.7 TGT treatment caused the immune dysfunction of liver lymphocytes

**Fig. 7:**
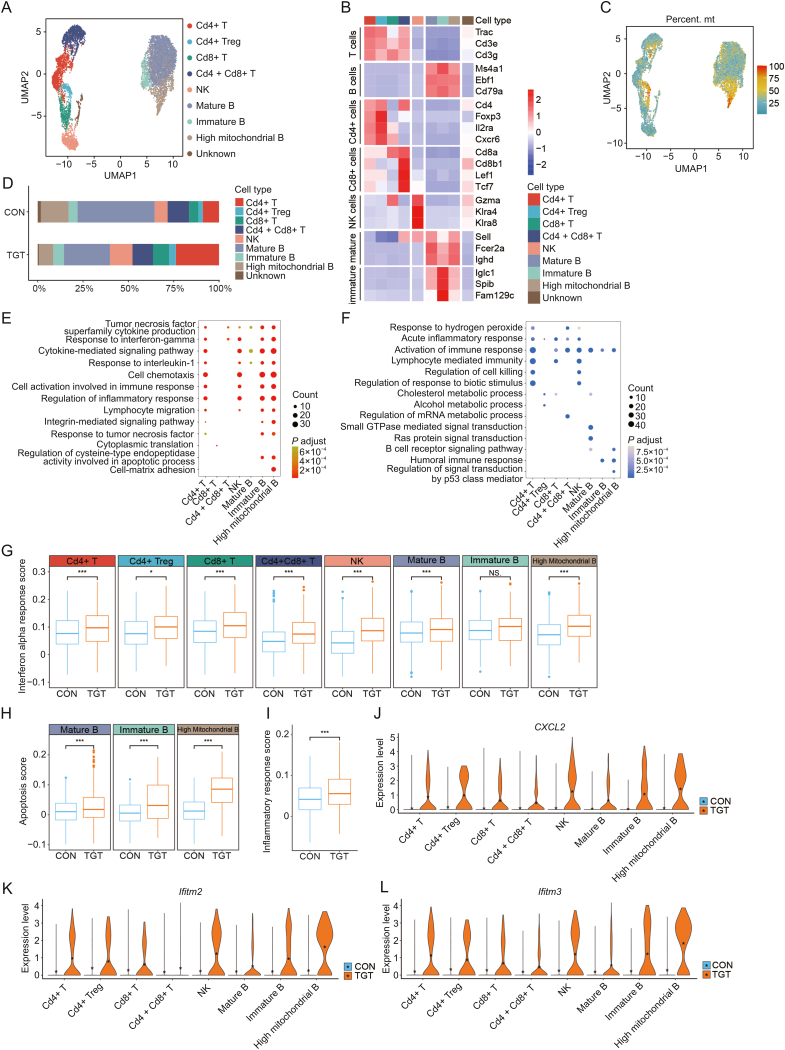
Function of lymphocytes was inhibited after tripterygium glycosides tablet (TGT) treatment. (A) Uniform manifold approximation and projection (UMAP) displays the subtypes of T, B, and natural killer (NK) cells. (B) UMAP displays the percent distribution of mitochondrial gene expression. (C) Heatmap plot shows the expression of subtype-specific genes for T, B, and NK cells. (D) Bar plot shows the fraction of T, B, and NK cell subtypes in the control (CON) and TGT groups. (E) Bubble plot represents the Gene Ontology (GO) enrichment analysis of upregulated differentially expressed genes (DEGs) in whole cell subtypes. (F) Bubble plot represents the GO enrichment analysis of downregulated DEGs in whole cell subtypes. (G) Functional comparisons of *IFN-a* response scores for all cell clusters between the CON and TGT groups. ^∗^*P* < 0.05, ^∗∗∗^*P* < 0.001, NS: not significant. (H) Functional comparisons of apoptosis scores for each B cell cluster between the CON and TGT groups. ^∗∗∗^*P* < 0.001. (I) Functional comparisons of inflammatory response scores for NK cells between the CON and TGT groups. ^∗∗∗^*P* < 0.001. (J–L) Violin plots show the expression of *CXCL2* gene (J), *Ifitm2* gene (K), and *Ifitm3* gene (L) for all cell subtypes in the two groups.

Lymphocytes play a critical role in immune regulation in the liver. In the current study, we delineated the subtypes of liver lymphocytes, including T cells, NK cells, and B cells, based on key gene markers. As shown in Fig. 7A, T cells were categorized into four subtypes, Cd4+ T cells (Cd4+ T), Cd4+ T regulatory cells (Cd4+ Treg), Cd8+ T cells (Cd8+ T), and Cd4+Cd8+ T cells (Cd4+Cd8+ T), while B cells were divided into mature B, immature B, and high mitochondrial B cells based on the specific expression genes as described in Fig. 7B. The mitochondrial genes with crucial cell processes (such as energy supply, signal transduction, apoptosis, and metabolic pathways) are significantly increased in the liver cells of hepatocellular carcinoma patients [26]. Interestingly, UMAP showed that the mitochondrial genes are mainly expressed in Cd4+ T and high mitochondrial B cells (Fig. 7C), indicating their regulatory effects on liver injury. In the case of liver diseases, immune cells can be regarded as an alarm system by increasing cytokine production and recruiting more immune cells to liver tissues as a protective mechanism. In this study, TGT treatment significantly increased the cell fractions of Cd4+ T and Cd8+ T compared to the control and decreased the proportion of mature B cells and high mitochondrial B cells, indicating that TGT activated immune cells to protect the body by triggering inflammatory responses to control inflammatory conditions but also lead to liver injury (Figs. 7D, and S6A–C). Also, GO enrichment analysis was performed according to the up- and down-regulated DEGs in the whole cell subtypes. As described in Fig. 7E, the upregulated pathways in the TGT treatment group were enriched in liver inflammation and apoptosis, which was consistent with previous findings on liver injury [27,28]. For downregulated DEGs, GO enrichment analysis revealed that TGT inhibited the activation of immune cells, cholesterol, alcohol, and RNA metabolism, further leading to acute liver injury (Fig. 7F). IFN-α is a type of cytokine secreted by immune cells with major functions, including broad-spectrum antiviral effects, immunoregulatory effects, and antitumor effects [29]. Compared to the control group, TGT treatment increased the *IFN-a* response score, which was closely related to the immune condition in all the eight subtypes of lymphocytes, indicating that TGT caused immune dysfunction of lymphocytes in the liver (Figs. 7G, S6D and S6E). Importantly, TGT led to a marked increase in the apoptosis score (Figs. 7H, and S6F) for three B cell subtypes and inflammatory response score (Figs. 7I, and S6G) for NK cells, which was in accordance with functional enrichment analysis results. C-X-C motif chemokine 2 (*CXCL2*), alternatively known as macrophage inflammatory protein 2, has the major function of chemokine leukocytes at the inflammatory sites. Interestingly, TGT treatment elevated the expression of *CXCL2* in all the liver lymphocyte subtypes, reflecting immune regulation (Fig. 7J). IFN-induced transmembrane proteins 2 and 3 (*Ifitm2, 3*) belong to IFITM proteins that are the cofactors for efficient virus infection in human cell types, including SARS-CoV-2 infection [30] and acute/chronic hepatitis C virus infection [31]. Thus, these proteins have been regarded as potential targets for therapeutic approaches to infections. In the current study, the liver tissues of mice in the TGT treatment group showed significantly higher expression levels of both *Ifitm2* and *Ifitm3* in all the subtypes of lymphocytes. In summary, under TGT-induced acute liver injury conditions, the liver lymphocytes demonstrated immune dysfunction.

### 3.8 TGT treatment disrupted intercellular crosstalk in the liver microenvironment

**Fig. 8:**
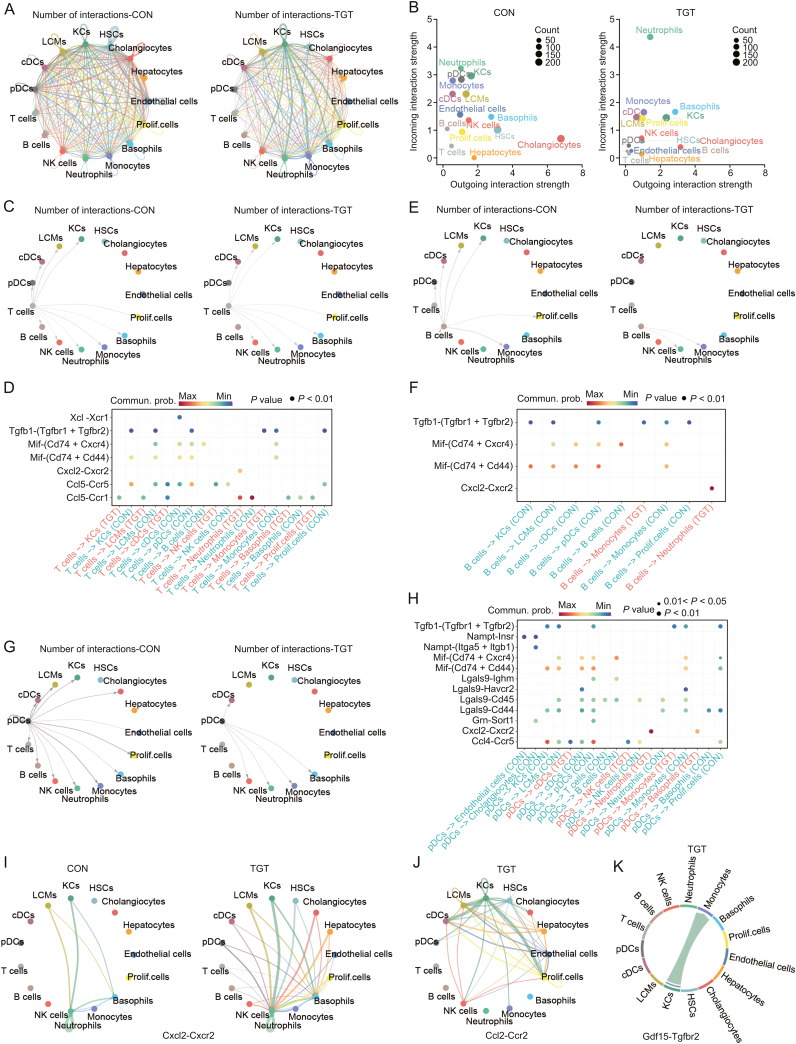
Tripterygium glycosides tablet (TGT) disrupted the intercellular crosstalk in the liver microenvironment. (A) Overall networks of cell-cell communication within different cell types for control and TGT groups. (B) Interaction strength of each cell type for control and TGT groups. (C) Chordal graph showing the differential interaction of T cells with other cell types. (D) Differential ligand-receptor interactions of T cells with other cell types. (E) Chordal graph showing differential interaction numbers of B cells interacting with other cell types. (F) Differential ligand-receptor interactions between B cells and other cell types. (G) Chordal graph showing the differential interaction between plasmacytoid dendritic cells (pDCs) interacting and other cell types. (H) Differential ligand-receptor interactions of pDCs with other cell types. (I) Interaction changes of *CXCL*2 and *CXCR2* after TGT treatment. (J) The *CCL*2-*CCR2* pair was discovered only after TGT treatment. (K) GDF (*Gdf15*-*Tgfbr2*) signaling pathway was observed only in the TGT group.

To deduce the intercellular interactions of TGT-induced acute liver injury, we constructed the cell-cell communication network within different cell types in liver tissues for the control and TGT groups via ligand-receptor (LR) pairs analysis between sender and receiver cells [32]. First, we calculated the number of cell-to-cell interactions among the 15 cell types in the liver tissues of both normal and TGT-treated mice and found marked differences between the two groups, as described in Figs. 8A and B. Also, the incoming and outgoing interaction strengths of various cell types, such as T, B, KCs, and HSCs varied greatly between the control and TGT groups. The global number and strength of almost all cell-cell interactions were reduced after TGT treatment, except the interactions of neutrophils (Fig. S7A). However, in this case, *CXCL* and *CCL* signaling pathways showed enhanced interactions compared to the control group (Fig. S7B). Next, we analyzed the crosstalk between T cells and the other 14 cell types. In the control group, T cells interact with B cells, NK cells, neutrophils, monocytes, basophils, Prolif. cells, pDCs, cDCs, LCMs, and KCs, whereas in the TGT treatment group, T cells did not show any interactions with B cells and pDCs (Fig. 8C). Thus, we identified 7 major LR pairs, which were differential ligand-receptor interactions for T cells interacting with other cell types, including *Tgfb1*-(*Tgfbr1* + *Tgfbr2*), *Mif*-(*Cd7*4 + *Cd44*), *CCL*5-*CCR5*, and *CCL5*-*CCR1*, indicating that T cells communicated with other cell types to regulate the inflammation and immune response (Fig. 8D). Similarly, in the normal liver tissues, B cells interacted with monocytes, Prolif. cells, pDCs, cDCs, LCMs, and KCs; whereas under TGT-induced acute liver injury, B cells did not interact with other cells except monocytes and increased interactions with neutrophils (Fig. 8E). In addition, we also found that TGT decreased the regulation of B cells on *Mif*-(*Cd74* + *CXCR4*), *Mif*-(*Cd74* + *Cd44*), and *CXCL2*-*CXCR2* indicating its inhibitory effects on regulation, as described in Fig. 8F. As shown in Fig. 8G, pDCs had interactions with all the other 14 cell types in normal liver tissue, whereas after TGT treatment, pDCs only showed interactions with cDCs, NK cells, neutrophils, monocytes, and basophils, further indicating the influence of TGT on the immune response. We also observed an enhanced interaction between pDCs and neutrophils/basophils via *CXCL2*-*CXCR2* in the TGT treatment group (Fig. 8H). In addition, compared to the normal group, TGT enhanced both the *CXCL* (Fig. 8I) and *CCL* (Fig. 8J) signaling pathway networks (Figs. S7C–E). Accumulating evidence indicates a pivotal role of the *CXCL2*-*CXCR2* signaling pathway in several biochemical processes, such as inflammation, immune response, angiogenesis, and wound healing [[33], [34], [35]]. The activated signaling facilitates neutrophil adherence to endothelial cells to facilitate migration into inflammatory sites [36]. Additionally, *CXCL2* expression can be regulated by various inflammatory mediators in endothelial cells, fibroblasts, melanocytes, monocytes, and megakaryocytes [37]. We also found that the expression of *CXCL2* was significantly upregulated in almost all cell types except neutrophils and basophils (Fig. S7F), which was further confirmed by ELISA. However, the expression of *CXCR2* was only elevated in neutrophils and basophils (Fig. S7F). These results were consistent with the cell-cell communication analysis. The findings implied that neutrophils might adhere and begin to migrate to the injured lesion after TGT treatment. Similarly, proinflammatory *CCL2* was highly expressed in endothelial cells, hepatocytes, HSCs, KCs, and LCMs (Fig. S7F), inducing an inflammatory response by binding to *CCR2*. Importantly, the GDF signaling pathway closely related to tissue injury was detected only in the TGT group.

### 3.9 TGT treatment significantly reduced the levels of inflammatory factors in mice

**Fig. 9:**
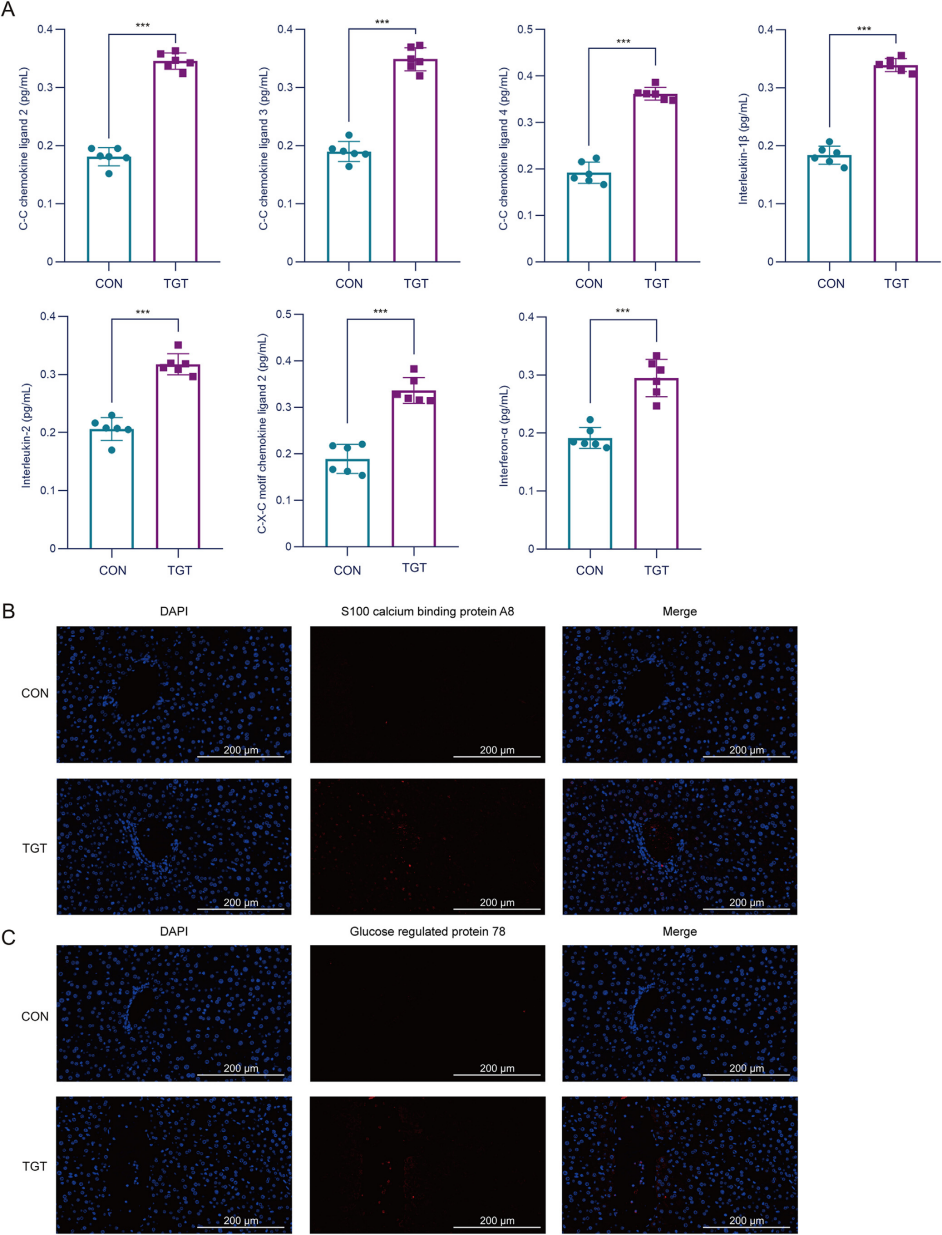
Single-cell sequencing results were verified in vivo. (A) Influence of tripterygium glycosides tablet (TGT) on the inflammatory cytokines in mice, *n* = 6. (B) Localization of S100 calcium binding protein A8 (*S100a8*, red) verified by immunofluorescence staining (40×). (C) Localization of glucose regulated protein 78 (*Grp78*, red) verified by immunofluorescence staining (40×). Data are expressed as the mean ± standard deviation (SD); ^∗∗∗^*P* < 0.001 vs. control (CON).

Our enrichment analysis of gene expression in different cells of the liver revealed that most were associated with inflammatory responses. These findings were confirmed by analyzing the key inflammatory factors and proteins. Compared to the CON group, the levels of *IL-1β*, *IL-2*, *CXCL2*, *CCL2*, *CCL3*, *CCL4*, and *IFN-a* of mice were markedly increased in the TGT group (Fig. 9A). Immunofluorescence staining results demonstrated a high expression of *S100a8* and *Grp78* in the TGT group (Figs. 9B and C). These findings were consistent with the results of single-cell sequencing analysis.

## 4 Discussion

While great efforts have been made to improve the application of TWHF-based therapy by investigating the mechanisms and manifestations of its toxicity, several critical gaps in knowledge still remain as barriers to the safe maximization of its therapeutic effects [38,39]. Based on the current clinical reports, TGT-induced liver damage is similar to that of acute viral liver injury [40]. Under injury conditions, the liver regulates the immune system by mediating bacterial clearance, cytokine, acute-phase protein production, and metabolic adaptation to inflammation [41]. In this study, we treated mice with TGT at a high dose (20-fold of the clinical equivalent dose) to induce acute liver injury, as described previously [6], and further validated the model by analysis of liver pathological structures and serum indices. Next, we constructed the first single-cell atlas of TGT-treated mouse liver and further analyzed the gene expressions and cell-cell communication crosstalk in several major cell types to illustrate the molecular and cellular events underlying TGT-induced acute liver injury. According to these findings, TGT treatment resulted in significant changes across different liver cell types, including liver endothelial cells, hepatocytes, HSCs, and LCM cells.

Liver endothelial cells, with their high capacity for endocytosis, maintain hepatic blood pressure and the quiescence of HSCs in physiological conditions [42]. However, in pathological conditions, liver endothelial cells become capillarized, losing their liver protective properties and promoting angiogenesis and vasoconstriction. Thus, they play a key role in the initiation and progression of several liver diseases, including acute or chronic liver injury, fibrosis, as well as hepatocellular carcinoma [43]. In this study, we identified seven subtypes of liver endothelial cells, which belong to the major cell types of LVEC and LSEC, with marked proportion changes between the control and TGT treatment groups. Further function annotations, gene enrichment analysis, and functional comparisons results showed that after TGT treatment, liver endothelial cells at different spatial locations undergo significant inflammatory responses through upregulated proinflammatory cytokines, such as *IL-1*β and chemokines, including *CCL 2/3/4*, *CXCL2*, and pro-fibrotic factors, such as several *S100* isoforms. Moreover, TGT increased the inflammatory response scores and EMT scores markedly. Thus, the dysfunction of liver endothelial cells contributed to the initiation and progression of TGT-induced acute liver injury.

Apoptosis is a key feature in several liver diseases. Among all the liver cells, hepatocytes are susceptible to apoptosis due to the common expression of death receptors. In acute liver injury conditions, hepatocyte apoptosis is the physiological route to maintain liver tissue homeostasis by eliminating damaged or infected cells [44]. In this study, we found that TGT treatment led to hepatocyte apoptosis in the major subtype of Hep1 with the primary functions of regulating fatty acid and amino acid metabolism. In contrast to that of normal livers, TGT significantly increased the fraction of Hep2 cells that are mainly focused on inflammation-related pathways, including cell chemotaxis and wound healing. The pseudotime trajectory analysis, which reconstructed cell-cell correlations, also indicated that the trajectory started with Hep1 cells, and most Hep3 cells progressed to Hep2 subtype. In this study, the influence of TGT on hepatocytes in acute liver injury conditions has been analyzed by both function enrichment and pseudotime trajectory analysis. These findings are in accordance with a previous study, which identified hepatocyte apoptosis and inflammation as novel mechanisms for the propagation of liver injury [45]. In summary, hepatocytes are related to the inflammatory response, apoptosis, and fatty acid metabolism dysfunction, which contribute to TGT-induced acute liver injury. The activation of HSCs and the consequent generation of myofibroblasts from activated HSCs lead to liver injury and fibrosis[[46], [47]]. Interestingly, HSC activation is further modulated by extracellular signals from resident as well as inflammatory cells, such as macrophages, LSEC, hepatocytes, NK cells, and B cells [48]. Based on this result, we speculated that TGT treatment significantly enhances the proliferation and activation of HSCs. The DEGs were mainly involved in the regulation of inflammation and immunity, which is consistent with the current understanding of the pathological mechanisms of liver injury [49]. Thus, our findings reinforced the remarkable complexity of HSC activation and also underscored the value of delineating the regulatory mechanisms for advancing the progress of novel treatments in TGT-induced acute liver injury.

As a distinct population of hepatic macrophages, LCM cells effectuate critical functions in the maintenance of hepatic homeostasis and the processes of acute and chronic liver injury [50]. In the present study, we used single-cell approaches to transform our understanding of the heterogeneity, dynamics, and functions of LCM cells in the livers of TGT-induced acute liver injury mice. Next, we identified four subtypes of LCM cells by different gene markers and found that TGT treatment significantly increased the fraction of LCM1 while markedly decreasing that of LCM4. In the functional regulation analysis, we showed that genes with altered expression after TGT treatment were closely involved in the regulation of the activation, inflammation, and phagocytosis of LCM cells, mediated by various cytokines, including *IL-6*, *CCL2*, and *CCL7*. Our findings are in accordance with published papers, which confirmed the contributions of activated LCMs in the liver under inflammatory conditions by regulating liver injury as well as liver fibrosis [[51], [52]]. According to the current knowledge, LCM cells extend dendrites into the blood as well as the peritoneal cavity and prevent the spread of peritoneal pathogens into the liver through neutrophils recruitment, and during this process, chemokine recruitment is crucial [53]. Furthermore, we found TGT increased the ER stress scores in all the LCM cell subtypes. Interestingly, *Grp78* is also known as the endoplasmic reticulum chaperone *BiP* and is markedly increased by TGT treatment in all the LCM cells, indicating that a possible mechanism underlying TGT-induced acute liver injury is ER stress [54]. The liver's lymphocyte population is mainly enriched in NK, T, and B cells. Next, we observed a sharp decrease in the total number of these cells after TGT treatment; detailed information is provided in the supplementary table. The significantly increased *IFN-a* response score in the eight subtypes of liver lymphocytes, the marked increase of inflammatory response score in NK cells, and the obvious increase in the apoptosis score in the three B cell subtypes suggested that TGT administration led to the immune dysfunction of liver lymphocytes. Given the importance of LCM cells and liver lymphocytes in regulating injury responses, we speculated that targeting LCM cells and liver lymphocyte subpopulations has the potential to function as a novel treatment strategy for TGT-induced acute liver injury.

Finally, according to the cell-cell communication crosstalk analysis, several cell types, such as T cells, B cells, KCs, HSCs, and pDCs play a central role in association with other cell types. Notably, in the liver tissues of TGT-treated mice, T cells showed maximal interactions with other cell types when acting as either source or target by ligand-receptor pairing. This included proinflammatory cytokines and the corresponding receptors, such as *Mif*-(*Cd74* + *Cd44*), *CCL5*-*CCR5*, and *CCL5*-*CCR1*, are closely involved in the innate immune response and the activation and amplification of liver injury [[55], [56]]. Reportedly, pDCs exert a protective role against immune-mediated acute liver injury [57]. In this study, we found that after TGT treatment, pDCs weakly interacted with other cell subtypes but had enhanced inflammatory ligand-receptor pairs. Thus, our finding was in line with reports that clarified the major role of immune cells in liver injury conditions [58].

Nevertheless, the present study has several limitations. Firstly, the molecular mechanisms underlying the regulation of different liver cell types under TGT-induced acute injury conditions need further *in vitro* investigation. Secondly, we analyzed the recruitment and activation of immune cells and found that both the clonal correlation and the environmental location of immune cells will require other techniques (such as spatial transcriptomics) to decipher the intricate activities of TGT in the liver fully. Finally, due to the complexity of the pathogenesis of TGT-induced acute liver injury, additional experiments are needed at the cell levels with single stimuli to validate the single-cell transcriptomic results.

In summary, this study integrated the in vivo experiments and single-cell RNA-seq, uncovering specific responses in different cell types and the microenvironment alterations in the liver tissues under TGT-induced acute liver injury conditions. These novel findings provided insights into the nature of TGT-induced acute liver injury and suggested potential pathways and targets for future therapeutic interventions to improve the safe and rational clinical applications of TGT.

## 5 Conclusions

In conclusion, we generated a comprehensive single-cell landscape for TGT-induced acute liver injury to identify the marked alterations and heterogeneity in 15 specific subtypes of liver cells under injury conditions. Importantly, our analysis expands our understanding of the mechanism of liver injury initiated by TGT, which would be valuable in identifying new biomarkers and therapeutic targets for liver protection. The present study also provided novel insights into the safe and rational usage of TGT in other therapeutic applications against rheumatoid arthritis, nephrotic syndrome, and other diseases.

## CRediT author statement

**Qiuyan Guo:** Conceptualization, Methodology, Resources, Visualization, Writing - Original draft preparation, Reviewing and Editing, Funding acquisition; **Jiangpeng Wu:** Software, Formal analysis, Investigation, Data Curation, Visualization, Writing - Original draft preparation, Reviewing and Editing; **Qixin Wang:** Validation, Formal analysis, Investigation, Visualization, Writing - Original draft preparation, Reviewing and Editing; **Yuwen Huang:** Investigation, Methodology, Software; **Lin Chen:** Investigation, Validation, Methodology; **Jie Gong:** Investigation, Writing - Reviewing and Editing; **Maobo Du:** Investigation, Writing - Reviewing and Editing; **Guangqing Cheng:** Investigation, Validation, Methodology; **Tianming Lu:** Investigation, Visualization, Validation; **Minghong Zhao:** Investigation, Visualization, Validation; **Yuan Zhao:** Software, Investigation, Resources; **Chong Qiu:** Investigation, Validation, Resources; **Fei Xia:** Investigation, Validation, Resources; **Junzhe Zhang:** Investigation, Validation, Resources; **Jiayun Chen:** Software, Methodology, Investigation; **Feng Qiu:** Supervision, Writing - Reviewing and Editing; **Jigang Wang:** Conceptualization, Resources, Supervision, Writing - Reviewing and Editing, Project administration, Funding acquisition.

## Declaration of competing interest

The authors declare that there are no conflicts of interest.

## Appendix A Supplementary data

Fig. S1 Quality control and cell type changes. (A) The number of genes for each cell for every sample (left) and two groups (right) after scRNA-seq data quality control. (B) The number of transcripts for each cell for every sample (left) and two groups (right) after scRNA-seq data quality control. (C) The percentage of mitochondrial genes for each cell for every sample (left) and two groups (right) after scRNA-seq data quality control. (D) The UMAP showing the sample distribution (left) and group distribution (right) corresponding to the 15 identified cell types. (E) The bar plot showing the fraction changes of each cell type for every sample (left) and two groups (right). Fig. S1

Fig. S2 Single-cell analysis of endothelial cells. (A) The UMAP showing the sample distribution corresponding to the identified endothelial cell subtypes. (B) The bar plot showing the fraction of endothelial cell subtypes in every sample. (C) Distribution comparison of endothelial cell subtypes from CON and TGT groups. (D) Venn diagram for up-regulated and down-regulated DEGs of endothelial cell subtypes after TGT treatment. (E) The functional comparisons of inflammatory response scores for endothelial cells between CON and TGT groups. ∗∗∗P < 0.001, ∗∗P < 0.01, ∗P < 0.05 (F) The functional comparisons of EMT scores for endothelial cells between CON and TGT groups. ∗∗∗P < 0.001, ∗∗P < 0.01, ∗P < 0.05. Fig. S2

Fig. S3 Single-cell analysis of hepatocytes. (A) The heatmap showing the expression of top 20 genes for each hepatocyte subtype. (B) The bar chart represents the GO enrichment analysis of every hepatocyte subtype. (C) The UMAP showing the sample distribution corresponding to the identified hepatocyte subtypes. (D) The bar plot showing the fraction of hepatocyte subtypes in every sample. (E-G) The functional comparisons of inflammatory response scores (E), apoptosis scores (F) and fatty acid metabolism scores (G) for hepatocytes between CON and TGT groups. ∗∗∗P < 0.001, ∗∗P < 0.01, ∗P < 0.05 (H) Pseudotime trajectory of hepatocyte subtypes based on cell subtypes. (I) The relative expression of specific genes accompanied with pseudo timing. (J) The violin plots showing the expression of S100a8, S100a9 and Clec4a genes for hepatocyte subtypes in the two groups. (K) The heatmap showing correlations of gene expression for hepatocyte subtypes. Fig. S3

Fig. S4 Single-cell analysis of HSCs. (A) The heatmap showing the expression of top 20 genes for each HSC subtype. (B) The UMAP showing the sample distribution corresponding to the identified HSC subtypes. (C) The functional comparisons of ROS scores for HSCs between CON and TGT groups. ∗∗∗P < 0.001, ∗∗P < 0.01, ∗P < 0.05 (D) The functional comparisons of inflammatory response scores for HSCs between CON and TGT groups. ∗∗∗P < 0.001, ∗∗P < 0.01, ∗P < 0.05 (E) Pseudotime trajectory of HSC subtypes. (F) The UMAP showing the distribution of HSC subtypes in the pseudotime analysis. (G) The heatmap showing top 30 genes involved in the differential development of cell fate1 (State 2) and cell fate2 (State 3). Fig. S4

Fig. S5 Single-cell analysis of LCM cells. (A) The bubble plot represents the GO enrichment analysis for every LCM cell cluster. (B) The UMAP showing the sample distribution corresponding to the identified LCM cell subtypes. (C) The bar plot showing the fraction of LCM cell subtypes in every sample. (D) The functional comparisons of phagocytosis scores for LCM cells between CON and TGT groups. ∗∗∗P < 0.001, ∗∗P < 0.01, ∗P < 0.05 (E) The functional comparisons of ER stress scores for LCM cells between CON and TGT groups. ∗∗∗P < 0.001, ∗∗P < 0.01, ∗P < 0.05 (F) Venn diagram for up-regulated DEGs of LCM cell subtypes after TGT treatment. (G) Venn diagram for down-regulated DEGs of LCM cell subtypes after TGT treatment. Fig. S5

Fig. S6 Single-cell analysis of T cells, B cells and NK cells. (A) The UMAP showing the sample distribution corresponding to the identified cell subtypes. (B) The bar plot showing the fraction of cell subtypes in every sample. (C) Distribution comparison of cell subtypes from CON and TGT groups. (D) The functional comparisons of interferon alpha response scores for T cells between CON and TGT groups. ∗∗∗P < 0.001, ∗∗P < 0.01, ∗P < 0.05 (E) The functional comparisons of interferon alpha response scores for B cells between CON and TGT groups. ∗∗∗P < 0.001, ∗∗P < 0.01, ∗P < 0.05 (F) The functional comparisons of apoptosis scores for B cells between CON and TGT groups. ∗∗∗P < 0.001, ∗∗P < 0.01, ∗P < 0.05 (G) The functional comparisons of inflammatory response scores for each identified cell cluster between CON and TGT groups. ∗∗∗P < 0.001, ∗∗P < 0.01, ∗P < 0.05. Fig. S6

Fig. S7 Cell–cell communication analysis for major cell clusters. (A) The number of inferred interactions and interaction strength for two groups. (B) The relative information flow between the two groups. (C) The change for CXCL signaling pathway network after TGT treatment. (D) The change for CCL signaling pathway network after TGT treatment. (E) Up-regulated and down-regulated signaling during TGT stimulation. (F) The violin plots showing the expression of CXCL2, CXCR2 CCL2 and CCR2 genes for all cell types in the CON and TGT groups. Fig. S7
