# Supplementary figures and images for: Single-cell transcriptome analysis uncovers underlying mechanisms of acute liver injury induced by tripterygium glycosides tablet in mice
Source: J Pharm Anal. 2023 Mar 22;13(8):908–25. doi: 10.1016/j.jpha.2023.03.004 (PMC10499593; doi:10.1016/j.jpha.2023.03.004)

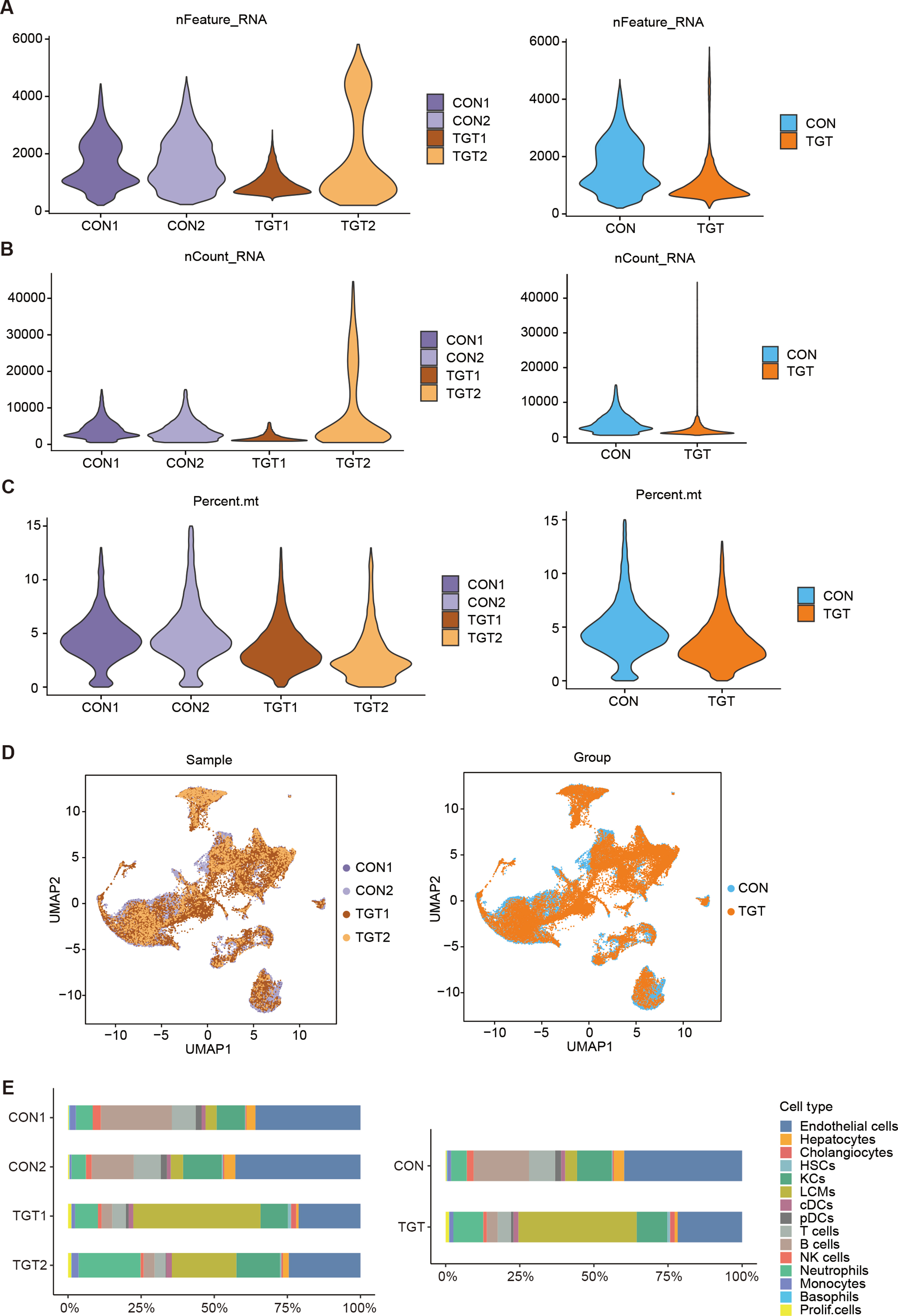

Supplement: Fig. S1 — Quality control and cell type changes. (A) The number of genes for each cell for every sample (left) and two groups (right) after scRNA-seq data quality control. (B) The number of transcripts for each cell for every sample (left) and two groups (right) after scRNA-seq data quality control. (C) The percentage of mitochondrial genes for each cell for every sample (left) and two groups (right) after scRNA-seq data quality control. (D) The UMAP showing the sample distribution (left) and group distribution (right) corresponding to the 15 identified cell types. (E) The bar plot showing the fraction changes of each cell type for every sample (left) and two groups (right). [file figs1.jpg]

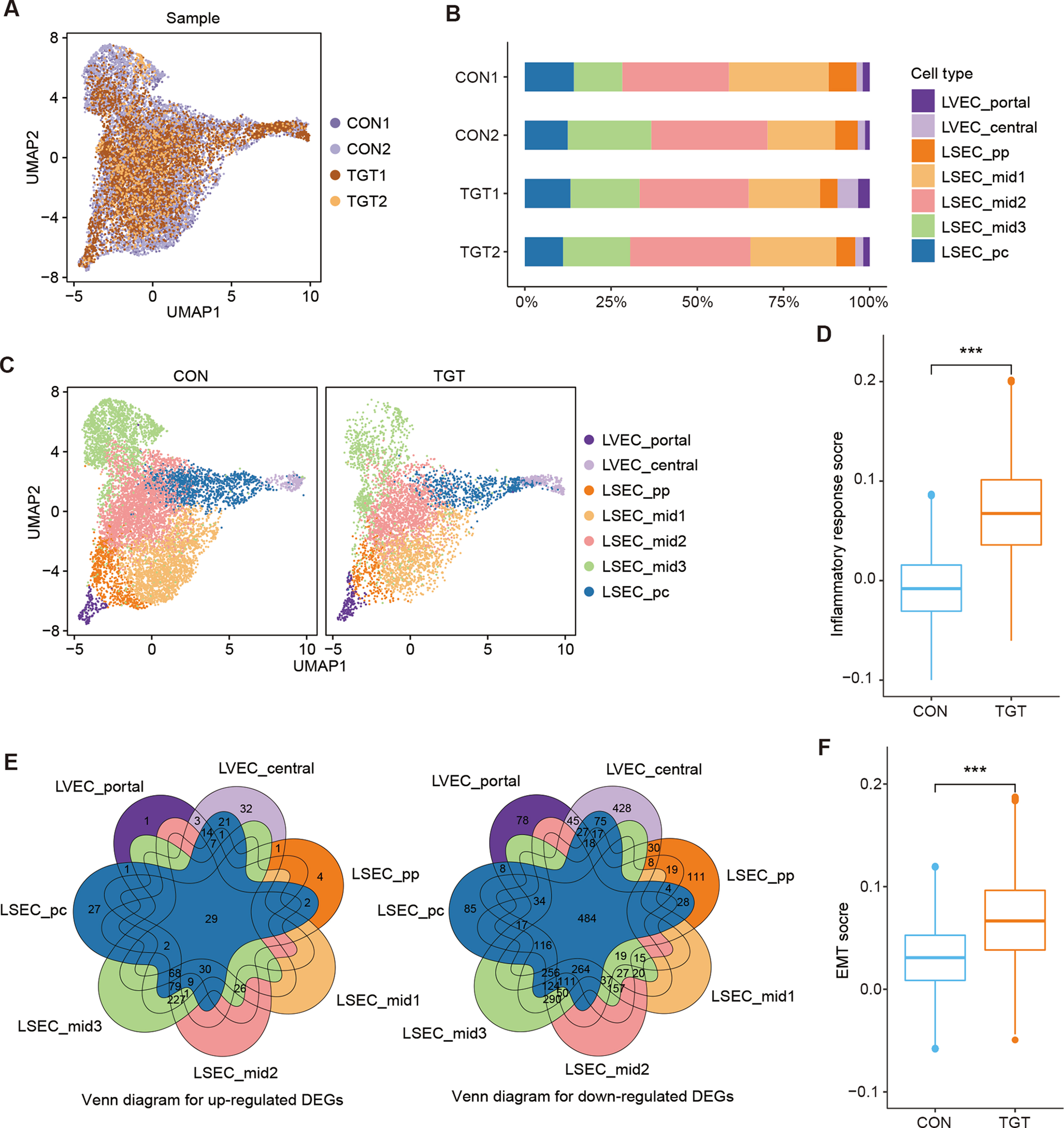

Supplement: Fig. S2 — Single-cell analysis of endothelial cells. (A) The UMAP showing the sample distribution corresponding to the identified endothelial cell subtypes. (B) The bar plot showing the fraction of endothelial cell subtypes in every sample. (C) Distribution comparison of endothelial cell subtypes from CON and TGT groups. (D) Venn diagram for up-regulated and down-regulated DEGs of endothelial cell subtypes after TGT treatment. (E) The functional comparisons of inflammatory response scores for endothelial cells between CON and TGT groups. ∗∗∗P < 0.001, ∗∗P < 0.01, ∗P < 0.05 (F) The functional comparisons of EMT scores for endothelial cells between CON and TGT groups. ∗∗∗P < 0.001, ∗∗P < 0.01, ∗P < 0.05. [file figs2.jpg]

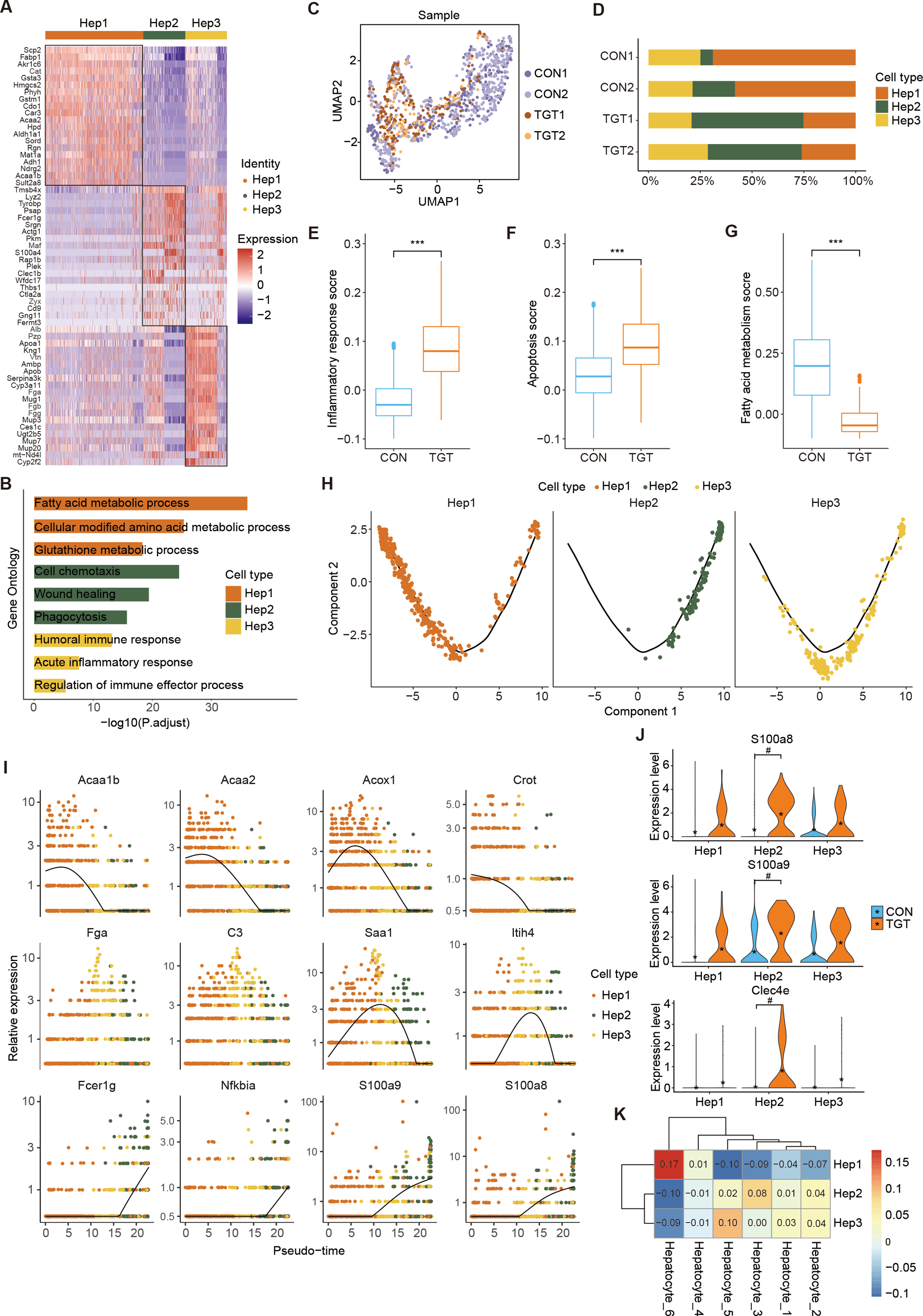

Supplement: Fig. S3 — Single-cell analysis of hepatocytes. (A) The heatmap showing the expression of top 20 genes for each hepatocyte subtype. (B) The bar chart represents the GO enrichment analysis of every hepatocyte subtype. (C) The UMAP showing the sample distribution corresponding to the identified hepatocyte subtypes. (D) The bar plot showing the fraction of hepatocyte subtypes in every sample. (E-G) The functional comparisons of inflammatory response scores (E), apoptosis scores (F) and fatty acid metabolism scores (G) for hepatocytes between CON and TGT groups. ∗∗∗P < 0.001, ∗∗P < 0.01, ∗P < 0.05 (H) Pseudotime trajectory of hepatocyte subtypes based on cell subtypes. (I) The relative expression of specific genes accompanied with pseudo timing. (J) The violin plots showing the expression of S100a8, S100a9 and Clec4a genes for hepatocyte subtypes in the two groups. (K) The heatmap showing correlations of gene expression for hepatocyte subtypes. [file figs3.jpg]

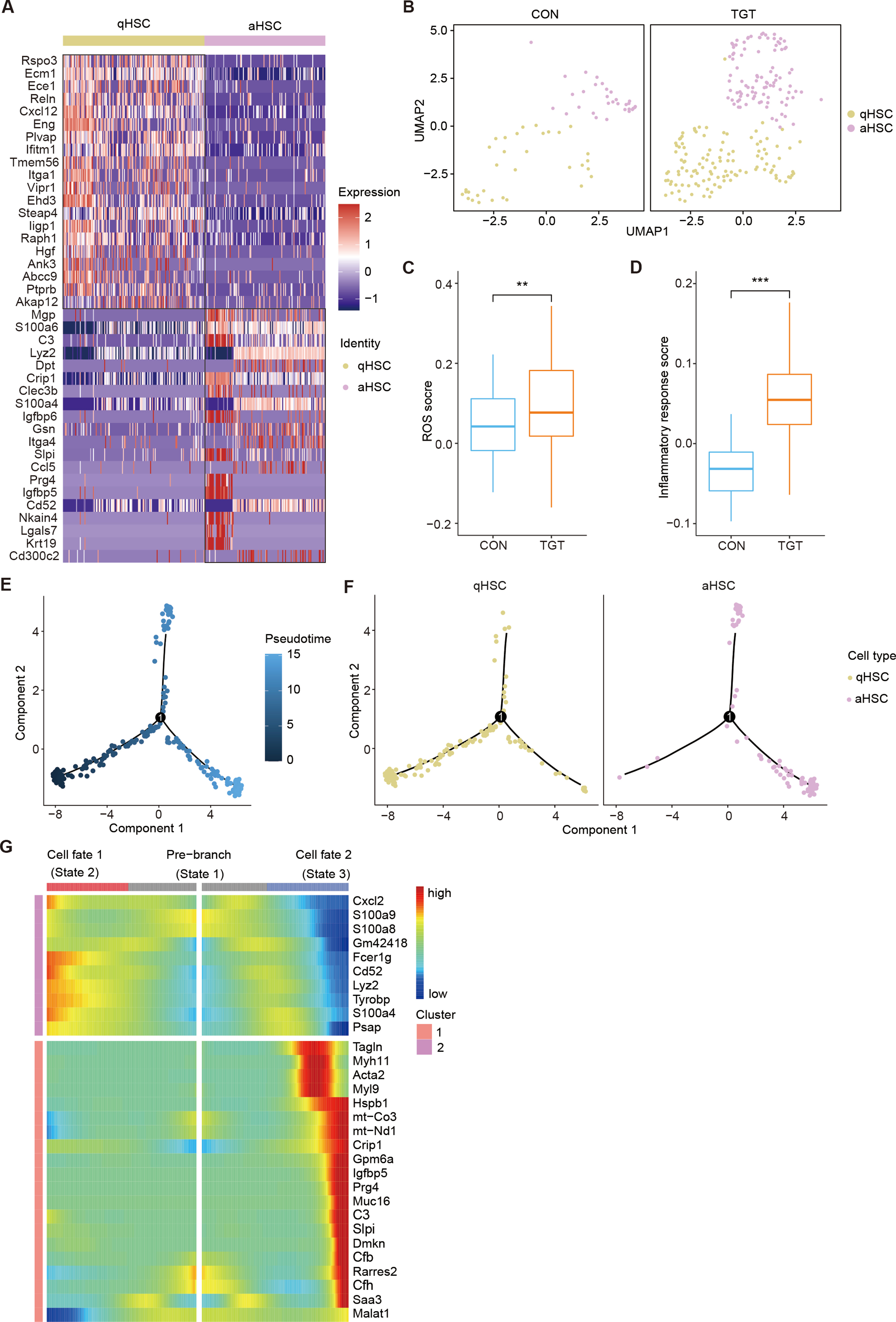

Supplement: Fig. S4 — Single-cell analysis of HSCs. (A) The heatmap showing the expression of top 20 genes for each HSC subtype. (B) The UMAP showing the sample distribution corresponding to the identified HSC subtypes. (C) The functional comparisons of ROS scores for HSCs between CON and TGT groups. ∗∗∗P < 0.001, ∗∗P < 0.01, ∗P < 0.05 (D) The functional comparisons of inflammatory response scores for HSCs between CON and TGT groups. ∗∗∗P < 0.001, ∗∗P < 0.01, ∗P < 0.05 (E) Pseudotime trajectory of HSC subtypes. (F) The UMAP showing the distribution of HSC subtypes in the pseudotime analysis. (G) The heatmap showing top 30 genes involved in the differential development of cell fate1 (State 2) and cell fate2 (State 3). [file figs4.jpg]

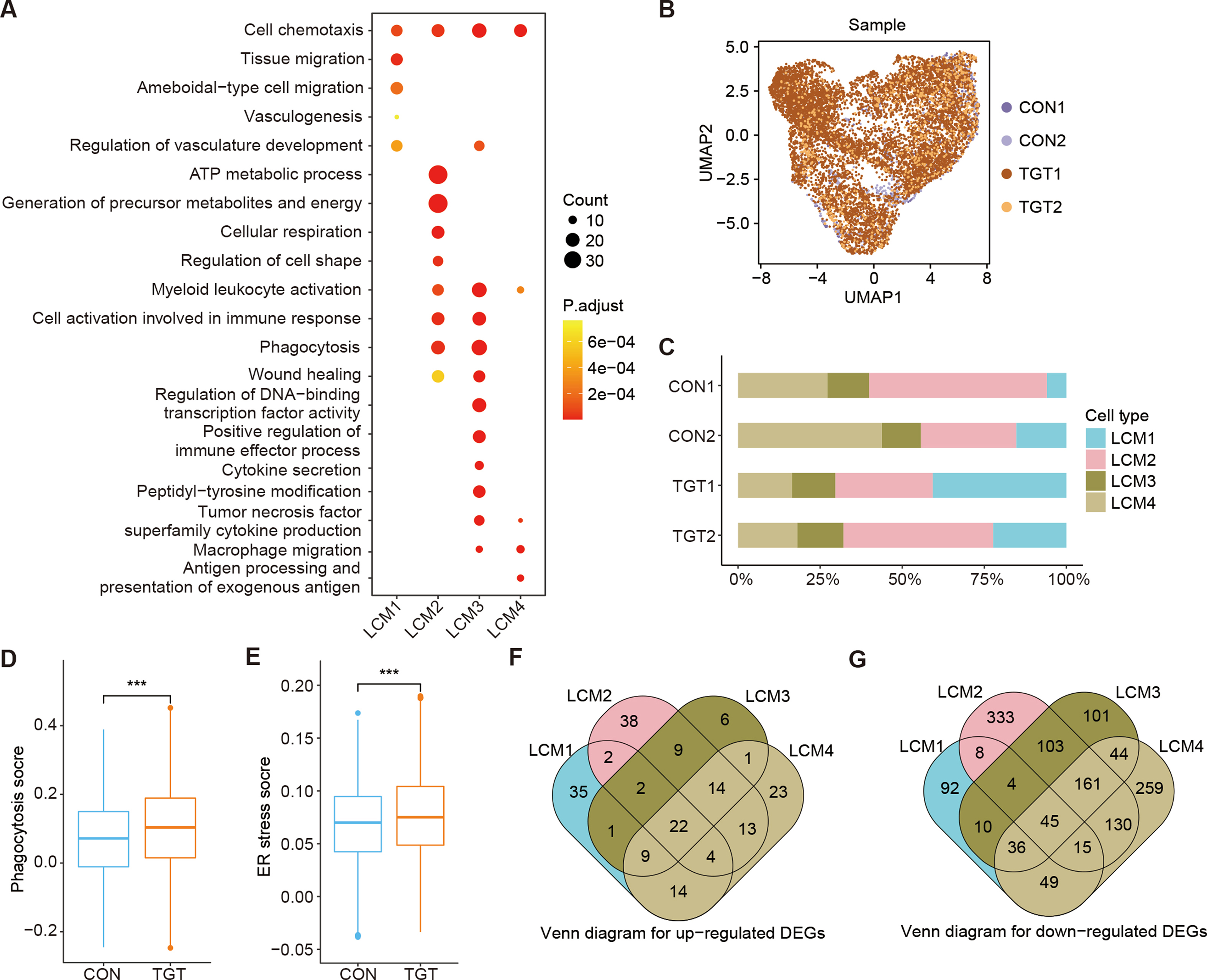

Supplement: Fig. S5 — Single-cell analysis of LCM cells. (A) The bubble plot represents the GO enrichment analysis for every LCM cell cluster. (B) The UMAP showing the sample distribution corresponding to the identified LCM cell subtypes. (C) The bar plot showing the fraction of LCM cell subtypes in every sample. (D) The functional comparisons of phagocytosis scores for LCM cells between CON and TGT groups. ∗∗∗P < 0.001, ∗∗P < 0.01, ∗P < 0.05 (E) The functional comparisons of ER stress scores for LCM cells between CON and TGT groups. ∗∗∗P < 0.001, ∗∗P < 0.01, ∗P < 0.05 (F) Venn diagram for up-regulated DEGs of LCM cell subtypes after TGT treatment. (G) Venn diagram for down-regulated DEGs of LCM cell subtypes after TGT treatment. [file figs5.jpg]

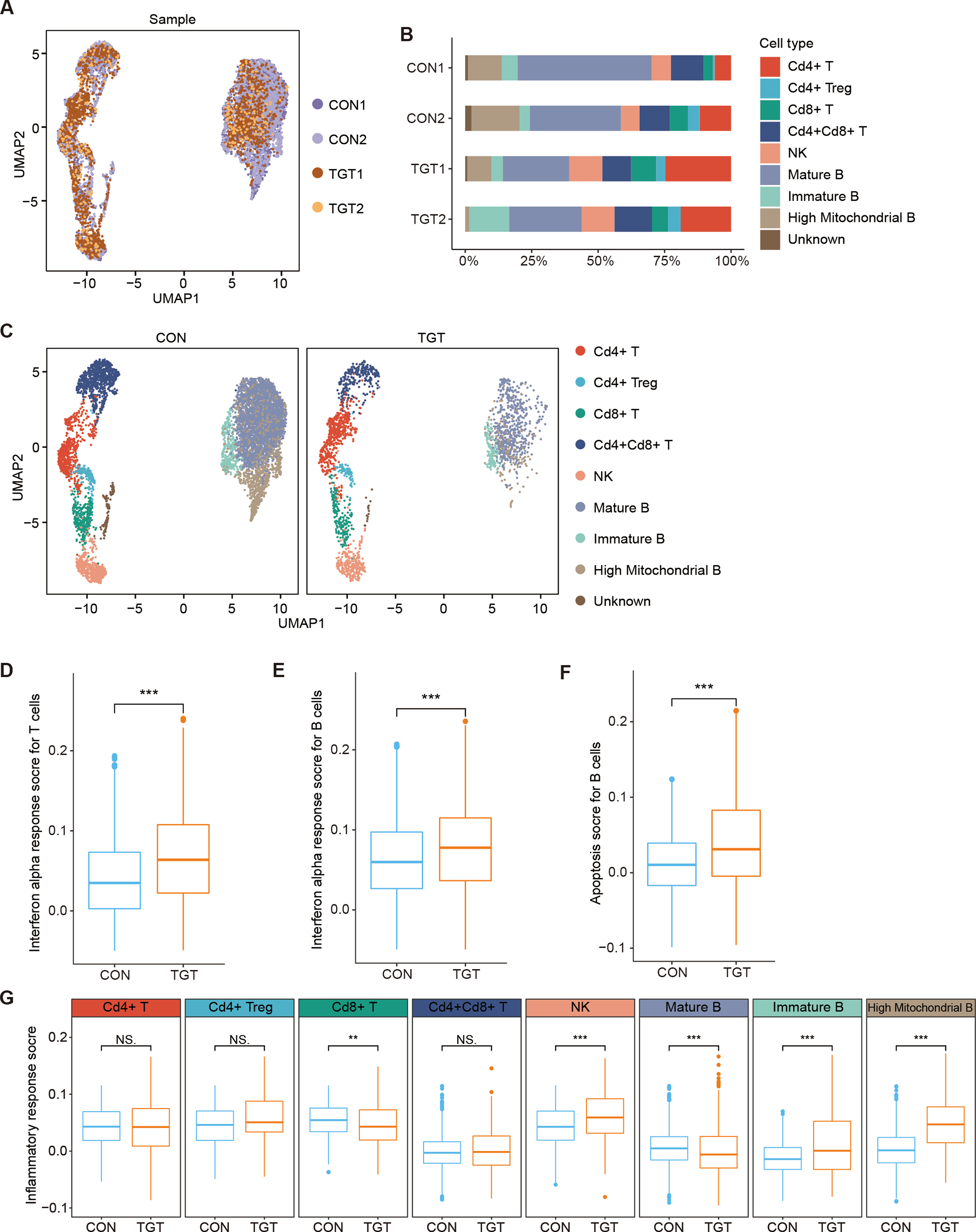

Supplement: Fig. S6 — Single-cell analysis of T cells, B cells and NK cells. (A) The UMAP showing the sample distribution corresponding to the identified cell subtypes. (B) The bar plot showing the fraction of cell subtypes in every sample. (C) Distribution comparison of cell subtypes from CON and TGT groups. (D) The functional comparisons of interferon alpha response scores for T cells between CON and TGT groups. ∗∗∗P < 0.001, ∗∗P < 0.01, ∗P < 0.05 (E) The functional comparisons of interferon alpha response scores for B cells between CON and TGT groups. ∗∗∗P < 0.001, ∗∗P < 0.01, ∗P < 0.05 (F) The functional comparisons of apoptosis scores for B cells between CON and TGT groups. ∗∗∗P < 0.001, ∗∗P < 0.01, ∗P < 0.05 (G) The functional comparisons of inflammatory response scores for each identified cell cluster between CON and TGT groups. ∗∗∗P < 0.001, ∗∗P < 0.01, ∗P < 0.05. [file figs6.jpg]

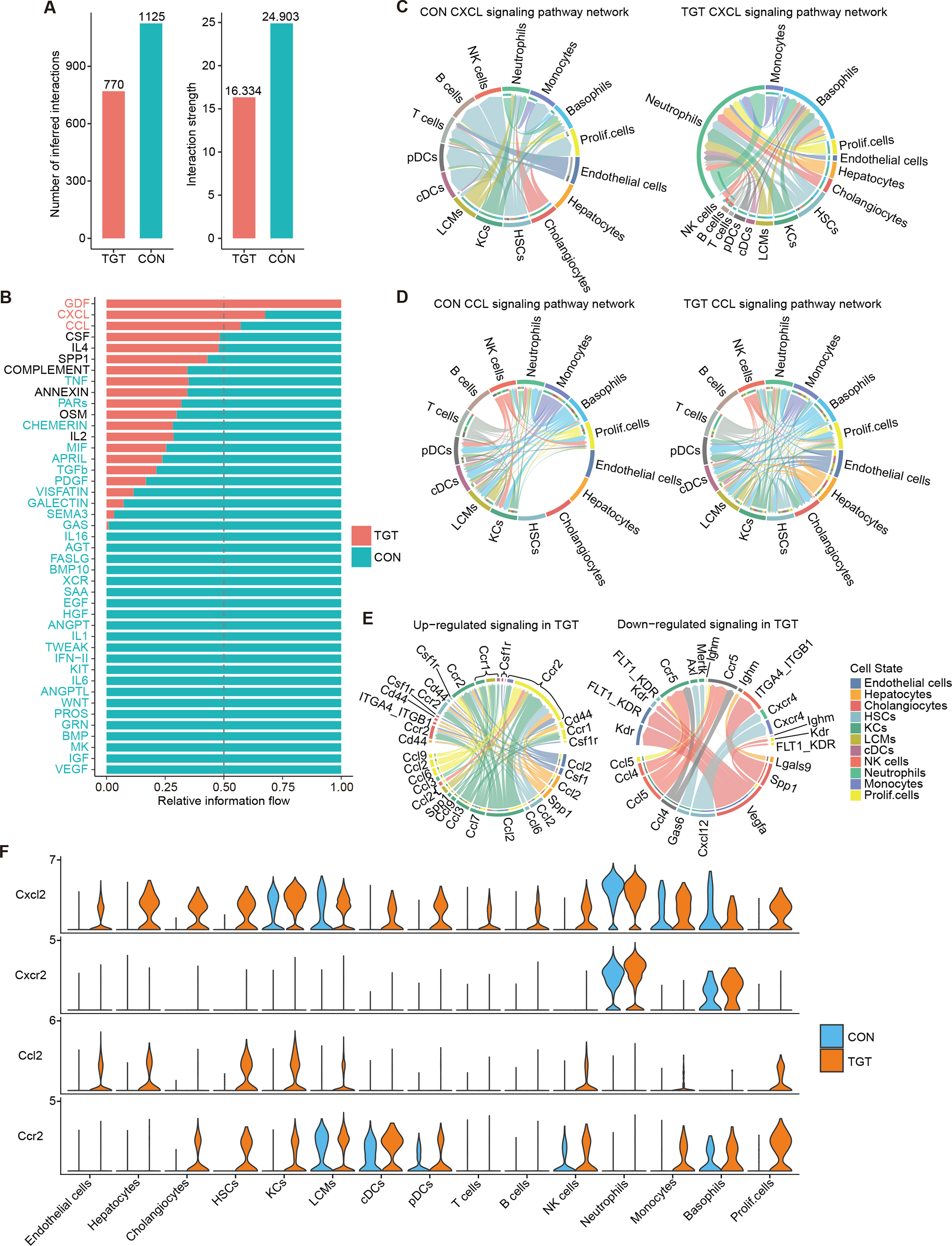

Supplement: Fig. S7 — Cell–cell communication analysis for major cell clusters. (A) The number of inferred interactions and interaction strength for two groups. (B) The relative information flow between the two groups. (C) The change for CXCL signaling pathway network after TGT treatment. (D) The change for CCL signaling pathway network after TGT treatment. (E) Up-regulated and down-regulated signaling during TGT stimulation. (F) The violin plots showing the expression of CXCL2, CXCR2 CCL2 and CCR2 genes for all cell types in the CON and TGT groups. [file figs7.jpg]
